# Public health informatics tools for dengue risk management: A systematic review

**DOI:** 10.1371/journal.pdig.0001495

**Published:** 2026-07-09

**Authors:** Naif M. Alraihan, Maddy French, Danielle Collingridge Moore, Luigi Sedda

**Affiliations:** 1 Lancaster Ecology and Epidemiology Group, Lancaster Medical School, Lancaster University, Lancaster, United Kingdom; 2 Public Health Authority, Riyadh, Saudi Arabia; 3 Division of Health Research, Lancaster University, Lancaster, United Kingdom; The University of Sheffield, UNITED KINGDOM OF GREAT BRITAIN AND NORTHERN IRELAND

## Abstract

Public health informatics (PHI) tools, including Geographic Information Systems (GIS), Electronic Health Records (EHRs), and Health Information Exchange systems, are increasingly applied to dengue fever surveillance, prevention, and control. Despite their growing adoption, a synthesis of empirical evidence examining their real-world application across endemic settings has not previously been conducted. This systematic review aimed to examine how PHI tools have been applied to dengue risk management, and to evaluate the certainty of evidence supporting their use. A structured literature search was conducted across PubMed, EBSCO/MEDLINE, and Web of Science. Nineteen peer-reviewed empirical studies published between 2010 and 2024 were included following eligibility screening against pre-defined inclusion and exclusion criteria. Study quality was assessed using the Newcastle-Ottawa Scale adapted for cross-sectional studies. Certainty of evidence was evaluated using the GRADE framework across five domains: risk of bias, inconsistency, indirectness, imprecision, and publication bias. Findings were synthesised narratively and organised into three functional categories: mapping and visualisation (n = 12), epidemiological insights (n = 5), and enhanced surveillance (n = 2). GIS was the most frequently used tool, consistently identifying dengue hotspots and supporting spatial dengue risk mapping across diverse geographic settings. EHR-linked health information systems supported epidemiological profiling and, in a limited number of studies, improved outbreak detection. Certainty of evidence was rated as very low across all three categories, reflecting the low evidence associated to observational study designs, methodological heterogeneity, and the uniform reporting of positive findings across all included studies. PHI tools show consistent descriptive utility in dengue surveillance across diverse settings. However, given the very low certainty of evidence, conclusions should be interpreted with caution. Gaps remain in high-burden regions including Sub-Saharan Africa and the Middle East. Standardised evaluation frameworks, broader geographic representation, and integration with emerging digital health technologies are needed to strengthen the evidence base. Systematic review registration: PROSPERO; registration number CRD42024572021.

## Author summary

Dengue fever is a growing public health concern, especially in tropical and subtropical regions. With limited treatment options, early detection and prevention are essential. This review examines how public health informatics (PHI) tools (such as digital maps, electronic health records (EHRs), and data-sharing systems) have been used to support dengue control efforts. Nineteen studies published between 2010 and 2024 were grouped into three categories: understanding disease trends, mapping outbreaks, and enhancing surveillance. Geographic Information Systems (GIS) were the most used tools for disease and outbreak mapping, while EHRs and data sharing systems provided promising results for real-time detection and response. However, high-burden regions like Sub-Saharan Africa and the Middle East were underrepresented in the selected studies. Our findings highlight the value of PHI tools in improving disease monitoring and call for more investment and research in underrepresented regions to strengthen global dengue prevention strategies.

## Introduction

Public health informatics (PHI) applies information systems and digital technologies to address population health needs. It integrates principles from epidemiology, public health, computer science, and data management to support the design and implementation of systems for collecting, analysing, and sharing health information. These systems enhance the planning, monitoring, and evaluation of public health interventions [1].

A core function of PHI is to ensure high-quality, interoperable data that can be used for timely and accurate decision-making. Operating at the population rather than individual level, PHI draws on diverse datasets including clinical records, laboratory data, demographic information, and environmental indicators, among others, to support disease prevention, outbreak preparedness, and health equity initiatives [2,3]. This integrated data environment enables rapid situational awareness and coordinated public health action.

A wide range of informatics tools contribute to PHI functions. Key examples include Electronic Health Records (EHRs), Health Information Exchange (HIE), Geographic Information Systems (GIS), and data visualisation platforms. EHRs as any electronic system that collects and stores patient or population health information for surveillance purposes [4,5], provide the data infrastructure through which disease notifications, hospitalisation records, and clinical outcomes can be monitored systematically [6,7]. HIE systems enhance data sharing across healthcare organisations, enabling faster detection of emerging threats [8,9]. GIS translate epidemiological data into spatial outputs, enabling the identification of transmission hotspots, the mapping of environmental risk factors, and the targeting of vector control interventions [10], while dashboards and visualisation tools make complex information accessible to decision-makers and the public [11]. Emerging technologies such as artificial intelligence (AI) and machine learning (ML) further strengthen public health capacity through predictive modelling and automated analysis [12].

These capabilities are particularly valuable for dengue fever, a rapidly expanding vector-borne disease that places nearly four billion people at risk globally [13]. Dengue transmission is shaped by climatic, environmental, and demographic factors, making timely detection and coordinated response essential. Public health agencies continue to face challenges in early case identification, prediction of outbreak dynamics, and allocation of resources [14]. Strengthening surveillance systems is therefore critical for monitoring incidence, identifying high-risk areas, and guiding control measures [15].

Despite the growing availability of informatics tools, their application to dengue surveillance and control remains uneven and underexplored. Differences in system design, data quality, and interoperability limit their potential for coordinated public health action. This systematic review examines how PHI tools have been used to manage dengue fever data within dengue prevention and control activities, evaluates their contribution to surveillance and intervention activities, and identifies opportunities to enhance their application in dengue-endemic settings. The review addresses a gap in the literature by consolidating current evidence on PHI applications and clarifying their real-world impact on dengue management.

## Methods

This review is a narrative synthesis of the role of Public Health informatics tools on the control of dengue fever disease. This review utilizes the systematic review approach for the search process and analysis of the literature. The review question is: “What is the role of public health informatics in enhancing surveillance of dengue fever?” The protocol for this review has been published on the International Prospective Register of Systematic Reviews (PROSPERO; registration number CRD42024572021).

### Search strategy

An initial scoping search was conducted in PubMed and Google Scholar to map the existing literature, identify gaps, and refine the inclusion and exclusion criteria before the formal systematic search. This iterative process informed the development of a structured search strategy aligned with the review objectives.

Search terms were developed around three core conceptual blocks: public health informatics tools, surveillance, and dengue. The PICO framework was used to define the key components of the research question and guide term selection (Table 1). Within each conceptual block, synonyms and related terms were combined using the OR operator, while the three conceptual blocks were linked using the *AND* operator to capture studies addressing the intersection of informatics tools, surveillance activities, and dengue. This approach was designed to enhance search sensitivity while maintaining high specificity.

**Table 1:** PICO framework outlining the key concepts guiding the systematic review. This table summarises the Population, Intervention, Comparison, and Outcomes (PICO) elements used to define the review focus on public health informatics tools for dengue surveillance and control.

Population	Dengue cases
Intervention	Public health informatics, which is the use of information and technology to analyse health data from healthcare facilities or communities, assists public health professionals and decision makers in developing plans and strategies to improve the health of the population and communities. Public health informatics tools include electronic health records (EHRs), health information exchange (HIE), data visualisation tools, and geographic information systems (GIS).
Comparison	N/A
Outcomes	Impact on surveillance of, response to, and control of the spread of dengue fever.

Systematic searches were conducted on 31 December 2024 across three electronic databases: MEDLINE, Web of Science, and PubMed. The search was restricted to studies published between January 2010 and December 2024 to capture contemporary literature on digital and informatics-based surveillance. The full database-specific search strategy, including search concepts, search terms, Boolean operators, and combined search structure, is provided in Table 2 to support reproducibility.

**Table 2:** Full database search strategy and Boolean logic applied across PubMed, EBSCO/MEDLINE, and Web of Science. This table presents the full search strategy and Boolean logic applied across databases. Search terms were grouped into three conceptual blocks: public health informatics, surveillance, and dengue. Terms within each block were combined using OR, while the blocks were combined using AND to identify studies relevant to dengue surveillance and public health informatics.

Database	Search Concepts	Search Terms/ Boolean Operators
PubMed	Public Health Informatics	(“public health informatics”) OR (“medical informatics”) OR (“population health informatics”) OR (“electronic health records”) OR (EHR) OR (“health information exchange”) OR (HIE) OR (“geographic information systems”) OR (GIS) OR (“data visualization”)
	Surveillance	(“epidemiology”) OR (“public health surveillance”) OR (“population surveillance”) OR (“epidemiological monitoring”) OR (“surveillance”) OR (“monitoring”)
	Dengue	(“dengue”) OR (“dengue fever”) OR (“severe dengue”) OR (“dengue virus”) OR (“vector-borne disease”)
	Combined Boolean Structure	(Public Health Informatics terms) AND (Surveillance terms) AND (Dengue terms)
EBSCO/ MEDLINE	Public Health Informatics	(“public health informatics”) OR (“electronic health records” OR “electronic medical records” OR EMR OR EHR) OR (“health information exchange” OR HIE) OR (“geographic information systems”) OR (“data visualization”)
	Surveillance	(“surveillance”) OR (“monitoring”) OR (“epidemiology”) OR (“population surveillance”)
	Dengue	(“dengue”) OR (“dengue fever”) OR (“dengue virus”) OR (“vector-borne disease”)
	Combined Boolean Structure	(Public Health Informatics terms) AND (Surveillance terms) AND (Dengue terms)
Web of Science	Public Health Informatics	(“public health informatics” OR “population health informatics” OR “health informatics” OR “electronic health records” OR EHR OR “health information exchange” OR HIE OR “data visualization” OR “geographic information systems” OR GIS)
	Surveillance	(“public health surveillance” OR “epidemiology” OR “surveillance” OR “population surveillance” OR “epidemiolog*” OR “monitoring”)
	Dengue	(“dengue” OR “dengue fever” OR “severe dengue” OR “dengue virus”)
	Combined Boolean Structure	(Public Health Informatics terms) AND (Surveillance terms) AND (Dengue terms)

All literature extracted from the electronic database searches were imported into EndNote reference management software [16], and duplicates were removed before screening. A single reviewer screened all titles and abstracts against pre-defined eligibility criteria, retaining any record with unclear or uncertain relevance for full-text review to minimise the risk of inappropriate exclusion. To further enhance the reliability of the screening process, a random sample of almost 30% of the screened records was independently verified by senior author (LS) against pre-defined inclusion and exclusion criteria. Full agreement was reached on all verified records, indicating consistency in the application of eligibility criteria throughout the screening process. Full texts of all potentially eligible records were then retrieved and assessed against the inclusion and exclusion criteria presented in Table 3. Studies that did not meet the eligibility criteria were excluded, and reasons for exclusion were documented (S1 Table). Only studies satisfying all eligibility criteria were included in the final review. This systematic review was reported in accordance with PRISMA 2020 guidelines (S1 PRISMA Checklist). The complete screening and selection process is illustrated in the PRISMA flow diagram (Fig 1).

**Table 3:** Inclusion and exclusion criteria used for study selection in the systematic review. This table summarises the eligibility criteria applied during screening, outlining the characteristics required for inclusion and the conditions leading to exclusion from the final systematic review.

Inclusion criteria	Exclusion Criteria
Evidence based on empirical research including peer-reviewed articles (quantitative, qualitative and mixed-methods studies)	Evidence not based on empirical research including grey literature and non-peer reviewed sources (e.g., government publication, book chapter, newspaper or magazine article, a website or blog post document published by government agency)
Studies that used public health informatics tools to surveillance, control and prevention dengue fever	Studies focused solely on dengue modelling such as statistical, mathematical, predictive, or forecasting models (including artificial intelligence and machine learning approaches), whether applied to simulated or real-world data.
Articles describing public or population health informatics tools (electronic health records (EHRs), health information exchange (HIE), and data visualisation tools, geographic information systems (GIS))	Studies focused on health communication and social media tools only.
Articles focusing on dengue fever.	Articles that focused on infectious diseases or vector borne diseases other than dengue fever
English-language articles.	Other languages rather than English.
Articles focused on humans or risk factors affecting humans.	Articles focused on animals or just the vectors (mosquitoes).

### Inclusion and Exclusion criteria

**Fig 1:**
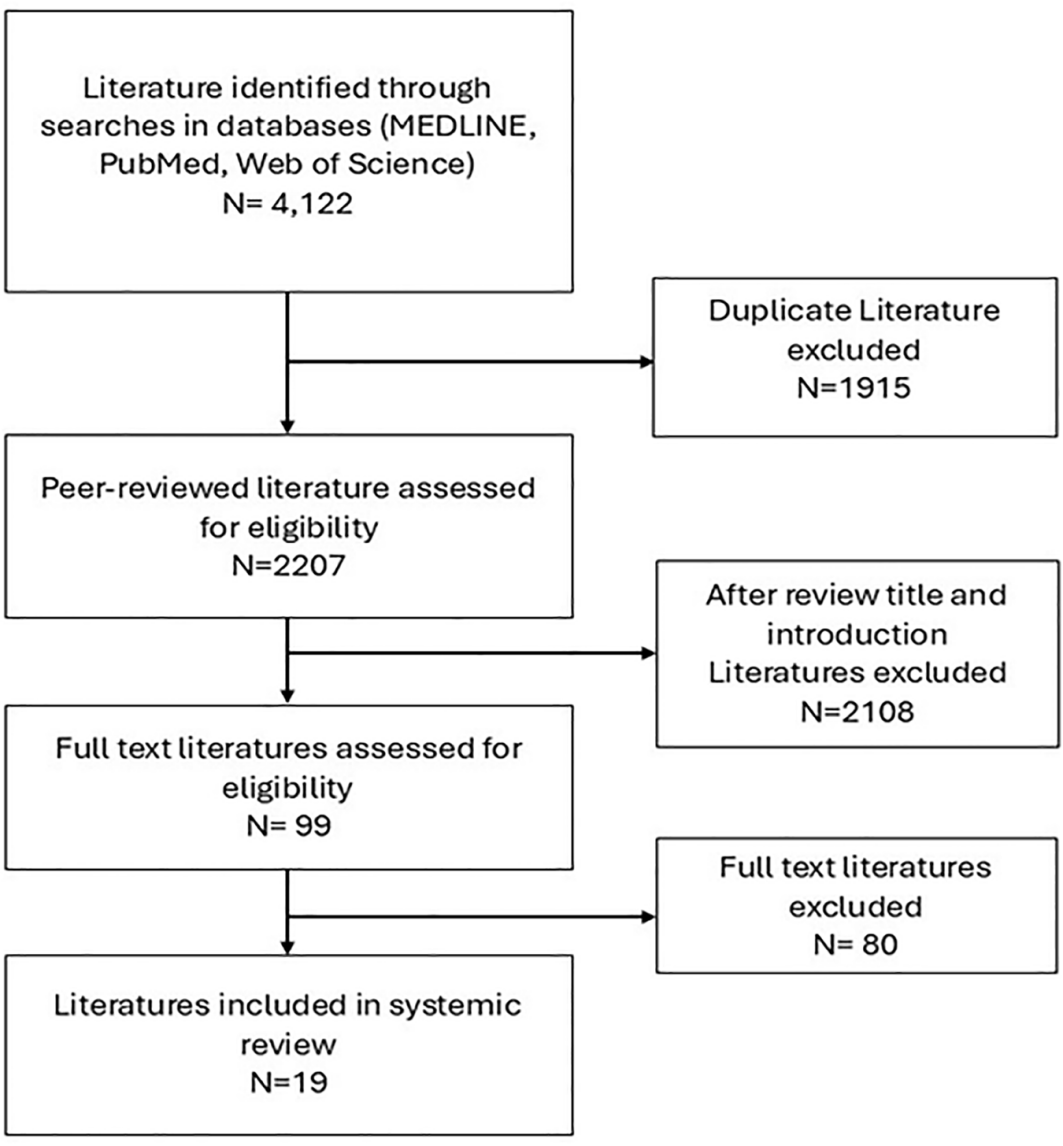
PRISMA diagram illustrating the study selection process. This figure illustrates the identification, screening, eligibility assessment, and final inclusion of studies in the systematic review. A total of 4,122 records were retrieved through database searches. After removing 1,915 duplicates, 2,207 records underwent title and abstract screening, resulting in the exclusion of 2,108 records. Ninety-nine full-text articles were assessed for eligibility, of which 80 were excluded based on predefined criteria. Nineteen studies met all inclusion criteria and were included in the final systematic review.

### Study selection criteria

Articles were included if they provided empirical evidence on the use of public health informatics tools for dengue case surveillance, data integration and management, mapping and visualisation of dengue clusters, and supporting responses to prevent or control the spread of dengue fever among human populations. Studies focusing on vector-borne or other infectious diseases, aside from dengue, were excluded.

This review focused specifically on PHI tools that had been operationally implemented or evaluated in real-world settings for dengue risk management, including surveillance, prevention, and control. Studies were excluded if their primary focus was the development, validation, comparison, or application of artificial intelligence, machine learning, statistical, mathematical, predictive, forecasting, or simulation models, unless these approaches were embedded within, implemented through, or evaluated as part of an operational PHI system. Therefore this criteria excludes studies that assess model performance, algorithmic accuracy, or theoretical transmission scenarios rather than the implementation, use, interoperability, or operational impact of PHI tools in public health practice [17–21]

In addition, studies that focused on animal populations and studies using health communication or social media-based surveillance were excluded, as these did not align with the review focus on formal PHI tools used in operational public health systems for dengue risk management [22].

### Quality appraisal and risk of bias

The quality of the included studies was assessed using the Newcastle-Ottawa Scale (NOS) for cohort and case-control studies, and an adapted version for cross-sectional studies [23]. The NOS served as both a quality appraisal and risk-of-bias tool for all included observational studies. Three domains were considered: selection of study groups, comparability, and outcome assessment. Studies were classified as having low, moderate, or high risk of bias according to their total NOS score. Results were tabulated for each included study.

### Data extraction

Data extraction was conducted using the JBI Mixed Methods Data Extraction Form, a standardised tool for retrieving key information from included studies. This covered publication details, study design and methodology, population and sample characteristics, the application of public health informatics tools, and key findings (S2 Table) [24].

### Reporting bias

Reporting bias was assessed by comparing each study’s stated objectives against its published results, following protocols from the Cochrane Handbook [25]. Each paper was examined for transparency regarding data limitations, including potential under-reporting and geographic constraints. Attention was given to whether authors disclosed non-significant findings or statistical anomalies, rather than reporting only positive correlations.

### Certainty of Evidence (GRADE)

The certainty of evidence was assessed using the GRADE framework [26], applied separately to three functional categories: mapping and visualisation (n = 12), epidemiological insights (n = 5), and enhanced surveillance (n = 2). As all included studies were observational, each category started at low certainty, consistent with GRADE conventions. Each category was then assessed for downgrading across five domains: risk of bias (drawing on NOS scores in Table 4), inconsistency of results, indirectness of evidence, imprecision of effect estimates, and likelihood of publication bias. Inconsistency was assessed by examining variation in study settings, target populations, informatics tools, and reported outcomes. Indirectness was considered in terms of alignment between included studies and the review’s question regarding real-world informatics use for dengue surveillance. Imprecision was judged against the number of studies and sample sizes within each category. Publication bias was assessed on the direction of reported outcomes, given that negative or null findings on informatics tool performance are underrepresented in this literature. Certainty was classified as high, moderate, low, or very low, following standard GRADE definitions [27].

**Table 4:** Quality appraisal and risk bias assessment of included studies using the Newcastle–Ottawa Scale (NOS). This table presents the risk of bias appraisal for all studies included in the final systematic review, assessed using the Newcastle–Ottawa Scale across three domains: Selection, Comparability, and Outcome. Total NOS scores ranged from 5 to 6, indicating that most studies demonstrated low to moderate risk of bias. Studies scoring 6–7 stars were classified as low risk, scores of 4–5 stars as moderate risk, and scores 0–3 stars as high risk.

	Selection			Comparability	Outcome			
	Representativeness of the sample	Selection of the non-exposed sample	Ascertainment of exposure	Comparability of outcome groups based on design of analysis	Assessment of outcome	Statistical test	Total stars	Total assessment score
Rehman et al.	*		*	**	*	*	6	Low risk of bias
Ng et al.	*		*	**	*	*	6	Low risk of bias
da Cruz et al.	*		*	**	*	*	6	Low risk of bias
Nor et al.	*		*	*	*	*	5	Moderate risk of bias
Gulley et al.	*		*	*	*	*	5	Moderate risk of bias
da Silva et al.	*		*	*	*	*	5	Moderate risk of bias
Faridah et al.	*		*	**	*	*	6	Low risk of bias
Baaji & Saravanabavan.	*		*	**	*	*	6	Low risk of bias
Abd Majid & Rasdi.	*		*	*	*	*	5	Moderate risk of bias
Mala & Jat.	*		*	**	*	*	6	Low risk of bias
Akter et al.	*		*	*	*	*	5	Moderate risk of bias
Atique et al.	*		*	*	*	*	5	Moderate risk of bias
Ahmad et al.	*		*	**	*	*	6	Low risk of bias
Chaiphongpachara et al.	*		*	**	*	*	6	Low risk of bias
Coelho et al.	*		*	*	*	*	5	Moderate risk of bias
Ahmad et al.	*		*	*	*	*	5	Moderate risk of bias
Hamer & Lichtveld.	*		*	*	*	*	5	Moderate risk of bias
Dom et al.	*		*	*	*	*	5	Moderate risk of bias
Jeefoo et al.	*		*	**	*	*	6	Low risk of bias

### Data synthesis

The collected data was synthesized using a narrative synthesis approach as outlined by Popay, Roberts [28]. This method has been chosen to take in account the anticipated heterogeneity of the data across the included studies. Heterogeneity is expected in terms of variations in participant demographics, differences in intervention approaches, diversity in outcome measures, and methodological differences among the studies. A narrative synthesis allows for a systematic yet flexible process of bringing together findings from diverse sources, enabling a comprehensive understanding of the research area.

The first step of a narrative synthesis involves constructing an explanation of how Public Health Informatics (PHI) functions, why it is effective in specific contexts, and who the primary beneficiaries are. Before the synthesis, a contextual understanding of PHI was developed, considering its role in public health interventions (see Introduction).

The second step of a narrative synthesis involves developing a preliminary synthesis of the collected data. This process examines how PHI has been applied across the included studies, particularly in relation to understanding dengue fever epidemiology, supporting disease control measures, managing and storing health data, visualizing information, and identifying risk areas through mapping. The synthesis focuses on key aspects of PHI implementation, including the technologies employed, data sources utilized, and their role in public health decision-making. Rather than assessing intervention outcomes, this stage highlights methodological variations, differences in PHI applications, and gaps in the existing research.

Building on this, the review then explores how PHI strategies differ across various contexts, particularly in their use for disease surveillance, risk assessment, and data visualization. By comparing findings from different studies, this analysis provides insights into how PHI has been adapted to regional needs and the factors influencing its effectiveness in dengue fever control efforts.

To ensure the reliability of the synthesis, a critical evaluation of the consistency of findings, methodological rigor, and coherence of the included studies is conducted. This assessment enhances the credibility of the conclusions drawn and provide valuable insights for future research and public health practice.

## Results

**Fig 2:**
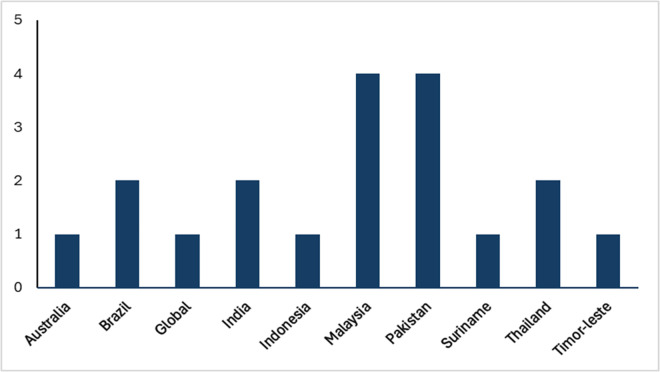
Geographic distribution of the 19 studies included in the systematic review. Malaysia (n = 4) and Pakistan (n = 4) were the most represented countries, followed by Brazil, India, and Thailand (n = 2 each). Single studies were conducted in Australia, Indonesia, Suriname, and Timor-Leste, and one study covered multiple countries.

Database searches identified 4,122 records (Fig 1). After duplicate articles removal and title and abstract screening, 99 full-text articles were assessed, of which 19 met the eligibility criteria (Table 5). Most studies originated from Asia, particularly Malaysia and Pakistan (n = 4 each), followed by India, Brazil, and Thailand (n = 2 each). Single studies were conducted in Indonesia, Suriname, Timor-Leste, and Australia, and one study included multiple regions [29]. Overall, the geographical distribution of the included literature was concentrated in Asia and South America, with additional studies from Australia and Suriname. No included studies were identified from Sub-Saharan Africa or the Middle East (Fig 2).

**Table 5:** Characteristics of the studies included in the final systematic review examining public health informatics tools for dengue surveillance and control. This table summarises the key characteristics of all studies that met the inclusion criteria for the final review, including study aims, settings, public health informatics (PHI) tools used, data sources, study designs, and main outcomes. The table forms the primary evidence base for the synthesis presented in this review.

Authors	Title Aim/Objective	Year	Country/population/ study duration	PHI Tools use	How were tools used?
Rehman et al.	Spatial mapping of dengue fever prevalence and its association with geo-climatic factors in Lahore, Pakistan Objective: To examine association between dengue fever prevalence and geo-climatic factors.	2024	Lahore, Pakistan 19882 dengue cases Between January and December 2021	GIS	• Mapping and Visualisation • Enabled targeted dengue control by identifying high-risk areas and liking outbreaks to environmental factors.
Ng et al.	Application of medical information system to identify dengue outbreak factors: Insights from a hyperendemic city in Malaysia Objective: To identify plausible factors causing dengue outbreaks in Ipoh, Malaysia.	2023	Ipoh, Malaysia 18812 number of participants From 2012 to 2019	EHRs visualisation tools GIS	• Epidemiological insights • Enabled the improved identification of outbreak risk factors and supports the control and prevention of dengue.
da Cruz et al.	Dengue in Timor-Leste during the COVID-19 phenomenon Objective: To assess dengue incidence and risk factors, including fatality rates, in relation to COVID-19 cases across Timor-Leste municipalities, while also evaluating how GIS can support stakeholders in understanding dengue prevalence, addressing challenges, and exploring development opportunities.	2023	13 Municipalities of Timor-Leste 1556 dengue cases From 2020 to 2021	EHR GIS	• Epidemiological insights • Played a pivotal role in identifying high-risk areas and assessing strategies to control dengue fever.
Nor et al.	Spatial and Temporal Analysis of Dengue Cases in Peninsular Malaysia; A Five-Year Analysis from 2016 to 2020 Objective: Used GIS to assess the prevalence of dengue cases and identify hotspots and cold spot regions in Peninsular Malaysia	2022	Regions in Peninsular Malaysia. 457968 dengue cases. From 2016 to 2020.	EHR GIS	• Mapping and visualisation • Visualises data to explore dengue distribution patterns, highlighting hotspots targeted by surveillance and intervention programmes.
Gulley et al.	A descriptive analysis of dengue in Peace Corps Volunteers, 2000–2019. Objective: To assess changes in incidence and data quality, analyse demographic and clinical outcomes using the enhanced data of the electronic medical record.	2021	East and South Asia, Caribbean, Central America, Pacific Islands, Africa, Europe and Central Asia. 1764 dengue cases. From 2000 to 2019.	EMR	• Enhance surveillance • Compared between the paper report (pre-EMR) and electronic record (post-EMR).
da Silva et al.	Profile of hospitalization and death records associated to dengue and severe dengue in Minas Gerais between 2000 and 2015 from the Brazilian Public Health System perspective. Objective: To evaluate the epidemiological impact of dengue fever and sever dengue.	2021	Minas Gerais State, Brazil 36330 dengue cases. From 2000 to 2015.	EHRs	• Epidemiological insight • Used to verify the relationship between the increase in dengue cases, high population density, and environmental factors.
Faridah et al.	Spatial and temporal analysis of hospitalized dengue patients in Bandung: demographics and risk. Objective: To assess the spatial distribution of relative dengue risk and identify high-risk groups by developing spatiotemporal patterns of dengue incidence, producing a map that visualizes relative dengue risk and highlights transmission hotspots to guide control strategies, and analysing demographic patterns (age, gender).	2021	151 villages of Bandung, Indonesia. 10573 dengue cases. From 2014 to 2016.	EHR GIS	• Mapping and visualisation • Visualises dengue risk areas based on demographic factors to assess distribution patterns across gender and age groups.
Balaji & Saravanabavan.	A geo medical analysis of dengue cases in Madurai city-Tamil Nadu India Objective: To map and analyse the spatial distribution of dengue cases, identify high-risk areas, analyse variations by age and gender, and guide effective dengue control measures.	2020	Madurai, India Use ward-wise distribution of dengue cases, out of 100 ward 85 wards reported cases in 2013, 83 wards reported cases in 2014, and 60 wards reported cases in 2015. From 2013 to 2015.	GIS	• Epidemiological insight • Utilized to identify disease patterns and implementing effective control measures.
Abd Majid and Rasdi.	Dengue Hotspot Detection in Bangi, Selangor, Malaysia. Objective: To detect and analyse dengue hotspots in order to understand spatial distribution patterns and assist in public health planning and disease control.	2020	Bangi city in Malaysia. 4507 dengue cases. From 2016 to 2019.	GIS	• Mapping and visualisation • Analyses spatial distribution to monitor and control dengue, facilitating public health interventions and resource allocation.
Mala and Jat.	Geographic information system based spatio-temporal dengue fever cluster analysis and mapping. Objective: To determine significant environmental and socioeconomic factors contributing to the patterns of dengue fever occurrences during the specified study period.	2019	Delhi, capital of India. 9483 dengue cases. From 2010 to 2012.	GIS	• Mapping and visualisation • Determines significant environmental and socioeconomic factors contributing to spatial-temporal clusters.
Akter et al.	Spatial and temporal analysis of dengue infections in Queensland, Australia: Recent trend and perspectives. Objective: To analyse the spatial and temporal trends of autochthonous and overseas-acquired dengue cases in order to identify high-risk areas and inform resource allocation for dengue control.	2019	Queensland, Australia 1773 dengue cases. From 2010 to 2015.	GIS	• Mapping and visualisation • Visualises high-risk areas to detect spatio-temporal trends and understand the seasonality of dengue cases.
Atique et al.	Investigating spatio-temporal distribution and diffusion patterns of the dengue outbreak in Swat, Pakistan. Objective: To investigate the spatio-temporal distribution, clustering, and diffusion patterns of the 2013 dengue outbreak in Swat, Pakistan, at the union council level and evaluate the influence of risk factors (population density, elevation, distance to river) on dengue incidence to guide control measures.	2018	Swat, Pakistan 9032 confirmed cases. Focused on the dengue outbreak from August to December 2013	GIS	• Mapping and visualisation • Investigates the spatio-temporal distribution by mapping hotspot clusters to develop surveillance and control strategies for dengue.
Ahmad et al.	Surveillance of intensity level and geographical spreading of dengue outbreak among males and females in Punjab, Pakistan: A case study of 2011. Objectives: To investigate the intensity levels and geographical spreading patterns of the 2011 dengue outbreak in Punjab, Pakistan, with a focus on differences between males and females across various age groups, using GIS-based epidemiological thematic mapping to identify hotspots and inform public health strategies.	2018	Punjab, Pakistan 21000 dengue cases. Focus on outbreak in various regions of the Punjab state in 2011.	GIS	• Mapping and visualisation • Visualises the link between demographic data that increase risk of dengue and hotspot locations to support control measures, resource allocation, and public health interventions.
Chaiphongpachara et al.	The Application of Geographic information system in dengue haemorrhagic fever risk assessment in Samut Songkhram province, Thailand. Objectives: To assess DHF risk and identify factors influencing DHF incidence by developing a GIS-based model to map risk areas at the provincial, district, subdistrict, and village levels, in order to support DHF surveillance and control, particularly for the protection of tourists and residents.	2017	Samut Songkhram, Thailand. 507 DHF cases in 2014 without document cases for 2015. From 2014 to 2015	GIS	• Mapping and visualisation • Integrates spatial and non-spatial data to designate risk areas for disease control and prevention.
Coelho et al.	Sensitivity of the Dengue Surveillance System in Brazil for Detecting Hospitalized Cases. Objectives: To evaluate the sensitivity of the Brazil’s national dengue surveillance system (SINAN) in detecting hospitalized dengue cases within the public health system (SUS) across 10 state capitals from 2008 to 2013, using probabilistic record linkage with SIH-SUS as the gold standard.	2016	10 state capitals, Brazil 71,161 hospitalized dengue cases from 2008 to 2013.	PHSS HIE	• Enhance surveillance • Evaluates dengue surveillance and response systems to support prevention and control strategies.
Ahmad et al.	Geographical Information System Based Approach to Monitor Epidemiological Disaster: 2011 Dengue Fever Outbreak in Punjab, Pakistan. Objectives: To map the geographical distribution and intensity of dengue cases, identify high-risk areas, track the spread of the disease over time, and demonstrate the utility of GIS in epidemiological surveillance and disaster management—particularly in resource-constrained developing countries like Pakistan.	2016	Punjab, Pakistan 21184 dengue cases Focus on the outbreak in 2011	GIS	• Mapping and visualisation • Evaluates the intensity of dengue in hotspot areas to develop precautionary measures.
Hamer and Lichtveld.	Spatial Distribution of Epidemiological Cases of Dengue Fever in Suriname, 2001–2012. Objectives: To characterize the frequency, incidence, and severity of dengue fever in Suriname, and to detect historical clusters of the disease by integrating epidemiological data into a spatial visualization platform.	2016	Suriname 2393 dengue cases. From 2001 to 2012.	GIS	• Mapping and visualisation • Identifies epidemiological clusters of dengue to implement preventive measures.
Dom et al.	Measurement of dengue epidemic spreading pattern using density analysis method: retrospective spatial statistical study of dengue in Subang Jaya, Malaysia, 2006–2010. Objectives: To analyse the spatiotemporal dissemination and identify hotspots of dengue outbreaks on a monthly basis, thereby enhancing the understanding and surveillance of the disease’s spread.	2013	Subang Jaya, Malaysia. 5,601 dengue cases. From 2006 to 2010.	GIS	• Epidemiological insight • Applied to linking dengue cases with mosquito populations to understand disease distribution and develop control and monitoring strategies.
Jeefoo et al.	Spatio-temporal diffusion pattern and hotspot detection of dengue in Chachoengsao province, Thailand. Objectives: To examine the spatiotemporal diffusion patterns of dengue fever and identify hotspots of reported dengue cases.	2011	Chachoengsao Province, Thailand. 5,831 dengue cases. From 1999 to 2007.	GIS	• Mapping and visualisation • Identifies the correlation and influence of risk factors on dengue transmission to understand disease trends, monitor outbreaks, and implement control measures.

### Methodologies

The 19 included studies employed a range of quantitative methods. Six ecological studies (e.g., da Cruz, Araujo [30]; Nor, Dom [31]; Balaji and Saravanabavan [32]) examined associations between dengue incidence and environmental or population-level factors. Five descriptive studies reported temporal trends and demographic characteristics of dengue cases, while three cross-sectional studies (e.g., Coelho, Leal [33]; Hamer and Lichtveld [34]; and Jeefoo, Tripathi [35]) assessed relationships between dengue incidence and demographic or environmental risk factors. Five retrospective observational studies (e.g., da Silva, de Andrade [36]; Faridah, Mindra [37]; Akter, Naish [38]) analysed long-term data series to evaluate surveillance performance, reporting completeness, and trends in disease burden. All studies relied on secondary data sources and were typically conducted at city or regional level, with only one study (da Cruz, Araujo [30]) conducted at national scale in Timor-Leste.

### Risk of bias, quality appraisal, and interpretation of evidence

All included studies were assessed using the Newcastle-Ottawa Scale adapted for cross-sectional studies, with total scores ranging from 5 to 6 out of maximum of 7 (Table 4). Nine studies scored 6 stars, indicating low risk of bias, and ten scored 5 stars, indicating moderate risk of bias. No study was rated as high risk of bias. Assessment covered three domains: selection, comparability, and outcome assessment. The most consistent limitation across studies was in the comparability domain, reflecting the descriptive and ecological nature of the included research, where few studies controlled for more than one confounder simultaneously.

These NOS evaluations informed the GRADE certainty of evidence assessment. Whilst no category was downgraded for risk of bias, the predominance of observational, retrospective, and ecologically descriptive designs using secondary surveillance, hospital, or spatial datasets meant that all three functional categories started at low certainty. The diversity of outcome definitions and tool applications across studies further limited direct comparison of findings and contributed to the inconsistency and indirectness identified in the GRADE assessment.

Reporting bias was assessed as low across all 19 studies. Authors generally reported weak or non-significant results alongside significant ones. Several studies explicitly noted the absence of a direct correlation between monthly rainfall and dengue incidence. Others reported that vegetation indices or wind speed did not significantly influence disease patterns in their settings. Most articles also acknowledged internal limitations, including likely under-reporting in national surveillance databases and limited spatial resolution in rural areas.

Using GRADE, all three functional categories started at low certainty, consistent with their observational designs. Mapping and Visualisation (n = 12) received two downgrades: indirectness, as most studies used retrospective secondary data rather than operational systems; and suspected publication bias, as no study reported null or failed outcomes. Epidemiological Insights (n = 5) received four downgrades: inconsistency across tools and outcomes, indirectness, imprecision given the small evidence base, and suspected publication bias. Enhanced Surveillance (n = 2) received five downgrade factors: inconsistency, indirectness, and very serious imprecision (two levels). All three categories were rated very low certainty (S3 Table).

### Types and use of PHI tools

**Fig 3:**
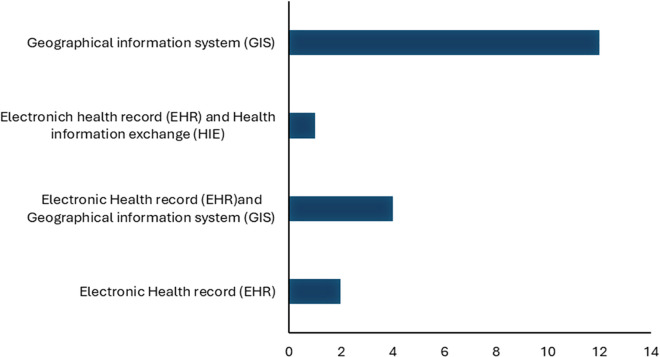
Frequency of public health informatics tools used across the 19 included studies. Geographic Information Systems (GIS) were the most widely applied tool (n = 12), followed by combined Electronic Health Records with GIS (EHR + GIS; n = 4), Electronic Health Records alone (EHR; n = 2), and Electronic Health Records with Health Information Exchange (EHR + HIE; n = 1).

The included studies identified several public health informatics tools used to support dengue surveillance and analysis. Across the reviewed literature, GIS was the most widely applied tool, used in 12 studies, followed by combined EHR and GIS systems in four studies, EHR-only applications in two studies, and EHR with HIE in one study, as shown in Fig 3. GIS was employed to map dengue incidence, identify hotspots, and examine spatial clustering in relation to demographic and environmental variables (Abd Majid and Rasdi [39]; Ahmad, Asif [40]; Ahmad, Asif [41]; Atique, Chan [42]; Dom, Ahmad [43]; Jeefoo, Tripathi [35]; Mala and Jat [44]; Rehman, Nasar-u-Minallah [45]). EHRs supported case identification, demographic profiling, and the analysis of clinical outcomes (Ng, Linus-Lojikip [46]; da Cruz, Araujo [30]), while HIE systems enabled standardised data exchange across institutions (Coelho, Leal [33], Rehman, Nasar-u-Minallah [45]). Data visualisation platforms facilitated rapid interpretation of spatial and temporal patterns, improving the representation of dengue trends (Ng, Linus-Lojikip [46]).

### Categorisation of PHI APPLICATIONS

**Fig 4:**
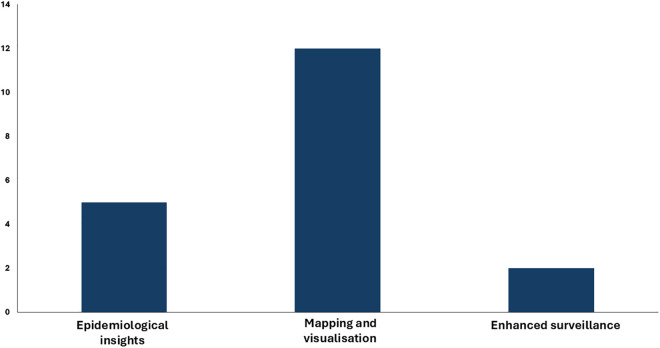
Categories of including literature based on rational used for public health informatics tools. Studies were grouped into three categories based on their primary use of PHI tools: epidemiological insights (n = 5), mapping and visualization (n = 12), and enhanced surveillance (n = 2).

The included studies were grouped into three categories based on their application of public health informatics tools: epidemiological insights, mapping and visualisation, and enhanced surveillance (Fig 4). Most studies fell within the mapping and visualisation category (n = 12), followed by epidemiological insights (n = 5), while enhanced surveillance was less frequently represented (n = 2).

The studies categorised under epidemiological insights applied PHI tools to describe dengue transmission patterns, identify associated risk factors, and characterise case distributions. Ng, Linus-Lojikip [46] integrated EHR, GIS, and a visualisation platform in Malaysia and reported that alternating wet and dry periods were more strongly associated with dengue outbreaks than rainfall alone. da Cruz, Araujo [30] used EHRs and GIS in Timor-Leste to examine urban dengue clusters and documented a high proportion of severe cases, including DHF and DSS. In Brazil, da Silva, de Andrade [36] compared the national notification system (SINAN) with hospital information system data (SIH/SUS) and reported underreporting of dengue cases, alongside observed associations with population density. Studies conducted in India and Malaysia [32,43] reported links between dengue incidence and environmental or household factors such as unsafe water storage, inadequate waste management, and rapid urbanisation.

The next group of studies examined the spatial distribution of dengue, using PHI tools to map case locations, identify spatial patterns, and display geographic variation across different settings. Studies in the mapping and visualisation category focused on the geographical representation of dengue case data across different cities and regions. All studies in this category utilised GIS, except for Nor, Dom [31] and Faridah, Mindra [37], who employed both GIS and EHRs to describe spatial and temporal patterns of dengue transmission. These two studies reported seasonal peaks during the rainy period and identified clusters of cases in areas characterised by high population density and rapid urban development.

Abd Majid and Rasdi [39] used GIS to document the clustering of dengue cases in relation to environmental and sociodemographic characteristics, including urban growth, high population density, and water storage practices. Atique, Chan [42] found that dengue cases were spatially clustered and observed spread from initial infection zones to surrounding areas. Chaiphongpachara, Pimsuka [47] identified spatially vulnerable zones using GIS, while Ahmad, Asif [40] reported that real-time hotspot visualisation supported more rapid identification of areas with increased case concentration. Rehman, Nasar-u-Minallah [45] analysed spatial distribution patterns and noted uneven case distribution, with concentrations in locations characterised by poor sanitation, standing water, and dense vegetation. Mala and Jat [44] observed shifts in dengue clusters over time, reflecting seasonal changes and local patterns of transmission.

Ahmad, Asif [41] reported higher dengue incidence among males and documented spatial clustering within urban centres. Hamer and Lichtveld [34] found that dengue cases were concentrated in urban and coastal districts and described periodic increases associated with the rainy season. Jeefoo, Tripathi [35] examined spatial and temporal dengue spread and observed that outbreaks typically originated in central urban areas extend to surrounding districts. Akter, Naish [38] identified seasonal expansion of dengue cases into new locations and reported that case distribution varied with climate, geography, and population movement.

Enhanced surveillance was reported in a small number of studies, focusing on systems designed to improve the detection and reporting of dengue cases. Gulley, Murphy [29] compared surveillance among Peace Corps Volunteers before and after the implementation of electronic medical records (EMRs) and reported an increase in documented dengue cases following the introduction of EMRs relative to paper-based reporting. Coelho, Leal [33] assessed the performance of Brazil’s Notifiable Diseases Information System (SINAN) by comparing it with the Hospital Information System (SIH/SUS) for hospitalised dengue cases. The latter study reported low sensitivity of SINAN in detecting hospitalised cases, indicating discrepancies between the two systems. Both studies focused on evaluating how surveillance platforms captured dengue-related information and reported differences between electronic and national reporting systems.

### Comparative synthesis of PHI tool contributions

The included studies varied considerably in their stage of development and operational context. Twelve of the nineteen studies were retrospective mapping exercises in which research teams extracted secondary case data from local or national health authorities and applied GIS to analyse historical outbreak patterns [32,34,35,38–45,47]. In none of these studies GIS tools were embedded in the routine workflows of the relevant health department.

The remaining seven studies are drawn on operational health information systems. Four were government-managed platforms: the e-Dengue system in Malaysia [46], the national HIS/EIS in Timor-Leste [30], the e-Notifikasi system across Peninsular Malaysia [31], and the Peace Corps EMR network [29]. Three studies used operational national or city-level databases, though the analyses were conducted as discrete research exercises rather than routine practice: Brazil’s SINAN and SIH-SUS [33,36] and the disease surveillance database of the Bandung city health office [37].

This distinction matters for interpreting the evidence. Studies using operational government systems were better placed to assess surveillance performance because the tools had been applied across defined populations over sustained periods. The two studies that directly assessed surveillance outcomes, Gulley, Murphy [29] and Coelho, Leal [33] both used established operational systems. This suggests that measurable improvements in surveillance performance are more likely to be detected and reported where informatics tools are institutionally embedded rather than applied in isolation for research purposes.

## Discussion

This systematic review included 19 studies that applied public health informatics tools to dengue surveillance and control across diverse epidemiological settings. GIS was the most frequently used tool, followed by combinations of EHR and GIS, EHR-only applications, and only few HIE systems. The studies fell into three main application categories: mapping and visualisation, epidemiological insights, and enhanced surveillance. Most them relied on secondary datasets and demonstrated low to moderate risk of bias. Twelve of the nineteen studies were retrospective mapping exercises not embedded in routine health department workflows, and only two studies directly evaluated surveillance system performance. Current PHI applications are regionally concentrated and dominated by spatial analysis approaches, with relatively few studies utilising integrated or real-time surveillance systems.

The GRADE assessment rated certainty as very low across all three functional categories. This was driven primarily by the observational nature of the study designs of all included studies, compounded by indirectness, inconsistency, and suspected publication bias. Very low certainty means that the true effect of PHI tools on dengue risk management outcomes may differ substantially from what the available studies suggest. No included study was assessed as high risk of bias (Table 4); evidence spanned both low- and moderate-risk categories, which supported the use of all included studies in the narrative synthesis whilst requiring cautious interpretation. The evidence supports the conclusion that PHI tools, particularly GIS, have been widely applied to dengue surveillance and have produced useful descriptive and spatial information. It does not support stronger claims about whether these tools independently improve dengue risk management, including reduction in transmission or measurable gains in surveillance sensitivity at a population level. The synthesis therefore emphasises the reported use and potential contribution of PHI tools to dengue risk management rather than asserting effectiveness.

Across the reviewed literature, GIS consistently emerged as a central component for visualising spatial and temporal patterns of dengue, identifying high-risk areas, detect clusters, and examine relationships between dengue occurrence and environmental, demographic, or geographic factors. By transforming diverse secondary data into clear spatial maps, GIS can help public health officials identify high-risk areas and plan targeted preventive measures, reinforcing its important role in dengue surveillance and environmental risk analysis [48]. These contributions are particularly useful in dengue-endemic settings, where transmission often varies between neighbourhoods, urban zones, and environmental contexts.

Studies reviewed by Sulistyawati and Fatmawati [48] showed that GIS applications have examined dengue distribution by age, gender, population, climate, and environment, including examples from Sri Lanka, Delhi, Thailand, Timor-Leste, Pakistan, Malaysia, and the Philippines. These studies show that dengue risk is often associated with rainfall, humidity, temperature, population density, demographic vulnerability, drainage conditions, and urban infrastructure [48]. More recent evidence also shows that GIS-based risk mapping and hotspot detection are among the most common applications in dengue surveillance, supporting targeted interventions such as vector control, larval source management, environmental sanitation, and resource allocation [49].

Direct evidence on the effect of GIS on dengue risk management outcomes remains limited. However, most included studies concluded that GIS provided practical value by identifying hotspot clusters, mapping high-risk areas, and guiding targeted prevention strategies across diverse settings [35,39,40,42,45]. This practical contribution is consistent with wider evidence showing that GIS supports public health decision-making by translating case and environmental data into spatial information that can direct vector control, larval source management, and resource allocation [48,49]. EHR and HIE systems serve different but complementary functions, supporting case documentation, reporting completeness, data linkage, and information sharing across health services, hospitals, laboratories, and public health agencies [30,33,46].

The smaller number of studies using EHR and HIE should not be interpreted as evidence that these tools are less valuable for dengue surveillance and control. Rather, it suggests that dengue-specific applications of these tools remain less frequently reported in the peer-reviewed literature. Evidence from wider infectious disease surveillance shows that electronic clinical and laboratory data can strengthen public health surveillance by improving reporting completeness, timeliness, interoperability, and the link between clinical care and public health action [50]. The European Centre for Disease Prevention and Control has noted that EHRs may improve the timeliness and completeness of infectious disease reporting whilst reducing the burden of manual reporting [51]. A systematic review by Kruse et al. [7] found that EHRs can support population health through improved surveillance, data management, and disease prevention, although challenges such as missing data and limited interoperability remain important barriers.

Evidence from other infectious diseases shows that EHR-based systems can improve surveillance timeliness, completeness, and use of routine clinical data. Electronic Support for Public Health used EHR data to identify tuberculosis cases and report two cases 12 and 36 days earlier than standard reporting systems [4,52]. EHR-based surveillance has also reduced underreporting by combining laboratory results, diagnosis codes, and medication orders, with Lyme disease incidence estimates reported to be four to seven times higher than traditional surveillance estimates [53]. EHR-based approaches have also supported monitoring of influenza, SARS-CoV-2, and respiratory syncytial virus [54,55].

In Indiana, an HIE-enabled intervention used prepopulated electronic notifiable disease reporting forms for several conditions, including chlamydia and gonorrhoea. The intervention increased provider reporting rates and improved completeness for several report fields, although improvements in timeliness were less clear [8].

For chlamydia cases, the intervention reduced the mean reporting lag by 2.7 days compared with matched controls, suggesting that HIE can improve the timeliness of communicable disease reporting when integrated into clinical and public health workflows. However, the same study found only a small and non-significant improvement in time to close cases, indicating that HIE benefits may depend on the specific surveillance outcome being measured [56].

HIE systems can therefore strengthen public health reporting when integrated into routine clinical workflows, but their effectiveness depends on careful implementation, data quality, and evaluation [57,58]. In the context of dengue, these properties are relevant because surveillance in endemic settings depends on linking clinical diagnoses, laboratory confirmation, hospital data, demographic information, and public health response activities across institutions, functions that remain fragmented in many of the countries represented in this review.

### Geographical diversity

The global distribution of studies highlights both strengths and disparities in PHI adoption. The review found considerable geographical diversity, covering both endemic and emerging regions affected by dengue fever. Most included studies were conducted in Southeast Asia, a region with one of the highest global burdens of dengue and home to over 52% of the world’s population at risk [45]. In Southeast Asia (Malaysia, Thailand, Timor-Leste, and Indonesia), tools such as GIS were applied to integrate environmental, climatic, and urbanisation-related data, illustrating how urban density, water storage, and sanitation influence dengue transmission [59].

In Asia (India and Pakistan), the emphasis was on demographic and epidemiological datasets, supporting the identification of vulnerable groups and seasonal dynamics [60,61]. In contrast, evidence from South America demonstrates how weaknesses in health information infrastructure limit the potential of PHI, with underreporting and poor integration affecting surveillance [62]. In Australia, the use of surveillance data to distinguish imported from locally acquired cases shows how PHI can extend beyond local monitoring to address global mobility and cross-border health risks [63]. A multi-regional perspective is provided through the Peace Corps Volunteer study, where the adoption of electronic medical records improved the timeliness and accuracy of reporting compared with paper-based systems, underscoring the role of digital platforms in strengthening surveillance capacity across diverse contexts [4]. These examples show that while PHI tools remain adaptable across settings, their effectiveness depends on integration with local systems and responsiveness to regional challenges, including demographic pressures, health system weaknesses, global mobility, and digital transformation.

Despite the geographical diversity of the included literature, several high-burden regions remain underrepresented, particularly Sub-Saharan Africa and the Middle East. This absence should not be interpreted as evidence that public health informatics tools are not used in practice, but rather as a likely reflection of wider surveillance, infrastructure, and publication gaps. In Sub-Saharan Africa, dengue epidemiology remains poorly understood because many countries lack comprehensive surveillance and reporting systems, meaning that estimates often rely on local prevalence studies rather than national surveillance data [64]. Wider evidence on disease surveillance in the region also shows persistent challenges in integrated surveillance systems, including limitations in reporting, data quality, laboratory capacity, digital infrastructure, analytical software, and trained personnel [65].

The lack of eligible studies from the Middle East may reflect comparable challenges. Digital health research in fragile and conflict-affected Middle East and North Africa (MENA) settings remains relatively limited, and stronger evaluation is needed to guide the design and deployment of digital health technologies [66]. Digital health adoption in Arab countries is also shaped by digital literacy, internet access, trust in online health information, and broader digital divides [67].

Saudi Arabia, where dengue fever is endemic [68,69], is underrepresented in the reviewed studies. Research efforts have traditionally prioritised biomedical fields such as diabetes, obesity, and cancer, with relatively limited attention to informatics [70]. Contributing factors include inadequate technical infrastructure, shortages of skilled informatics professionals, limited cross-sector collaboration, and uneven funding that often leaves public health informatics under-supported [71].

The exclusion of non-English publications may also have contributed to the observed geographical pattern [72]. Dengue affects many countries where research and operational reporting may be published in Spanish, Portuguese, French, Arabic, or regional Asian languages [73]. This is particularly relevant for PHI because surveillance systems are often developed and used within national or regional public health agencies, and their implementation may be reported locally rather than in international English-language journals and peer-reviewed [74–76].

This lack of representation from high-risk regions limits global understanding of dengue burden and may restrict the development of context-specific surveillance and response strategies. Strengthening dengue-related PHI in underrepresented regions requires investment not only in digital platforms, but also in surveillance infrastructure, laboratory capacity, workforce training, and cross-sector collaboration [65,66,71]. This geographical gap should be interpreted cautiously, as it may reflect limitations in surveillance capacity, language coverage, database indexing, and publication practices rather than a true absence of PHI activity.

### Methodological diversity in PHI application

PHI tools were applied to evaluate the influence of diverse risk factors, including human activity, climatic conditions, and vector-related dynamics, on the incidence and transmission of dengue [50,77,78]. The breadth of methodological approaches employed across the included studies introduces limitations that should be considered when interpreting the findings or this review.

The included studies drew on data sources that varied considerably in their nature and origin, including national surveillance systems, hospital records, notification systems, electronic medical records, municipal surveillance data, and GIS-based environmental datasets [29–31,36,45,46]. These sources differ in how cases are recorded, confirmed, and reported, which limits direct comparison of findings across settings. Surveillance systems also varied between countries in their data capture procedures, diagnostic capacity, and reporting thresholds, introducing further variation that is independent of the PHI tools being applied.

Analytical approaches differed across studies and included descriptive epidemiology, GIS-based spatial mapping, hotspot detection, cluster analysis, temporal analysis, regression-based methods, and record linkage [33,35,38,44,46]. These methods were applied using different spatial units, time periods, covariates, and outcome measures, which limit the extent to which findings can be attributed to tool performance rather than methodological differences between studies.

Whilst this review focused on traditional research designs, it is important to recognise the increasing relevance of advanced analytical methods. In particular, AI and ML are emerging as promising approaches for predictive modelling, outbreak detection, and real-time disease surveillance [79]. Although studies involving AI and ML were excluded from this review, future research should explore their integration with existing PHI systems [80]. These technologies have the potential to complement tools such as GIS and EHRs, enhancing early warning systems and improving the precision of public health interventions [50,77].

### Framework for interpreting PHI contributions to dengue surveillance

This review grouped the applications of PHI tools into three broad categories: epidemiological insights, mapping and visualisation, and enhanced surveillance. This classification does not claim to provide a definitive or superior assessment of PHI tools but serves as a practical organising framework to interpret the diverse ways in which these tools have been applied to dengue surveillance and control. By aligning functions rather than focusing solely on individual technologies, the framework highlights how PHI contributes across different stages of public health practice.

These categories could be aligned to the six keys of public health functions identified by ECDC in 2021 [81]. Tools classified under enhanced surveillance category support functions such as surveillance and monitoring, outbreak detection and communication. The epidemiological insights category corresponds to outbreak response, disease pattern and the relationship between risk factors and dengue distribution. Mapping and visualisation support outbreak detection and response by enabling public health officials to identify high risk geographic areas.

GIS integrates spatial analytics, and data visualisation to represent health information in ways that support risk identification and decision-making [82]. Across the included studies, GIS was used to identify spatial clusters of dengue cases, detect hotspots, and map high-risk zones to support targeted planning. This pattern is consistent with wider evidence from Salim, Satoto [80], who identified GIS as the most dominant digital health intervention in dengue surveillance, accounting for 30% of all digital tools applied across their included studies.

### Integration for enhancing epidemiological insights

Many studies emphasised the importance of integrating multiple platforms to enhance disease monitoring and control. As highlighted by Williams, Oke [83], combining tools such as EHRs and GIS can strengthen public health responses and support the identification of key determinants of disease patterns. Their work underscores how integrating clinical and spatial data facilitates the recognition of epidemiological trends and environmental risk factors. The studies by Ng, Linus-Lojikip [46] and da Cruz, Araujo [30] illustrate how integration improves detection of seasonal, urban, and socio-economic influences, enabling more targeted interventions. These insights are essential for understanding risk dynamics and for designing more targeted interventions and control strategies to mitigate the spread of dengue fever.

Whilst only a small number of studies in this review explicitly focused on real-time data sharing and rapid detection, this area holds considerable potential for enhancing surveillance. Coelho, Leal [33] and Gulley, Murphy [29] both illustrate how EHR-based systems can improve clinical outcome tracking, support more complete case reporting, and reduce reliance on paper-based or fragmented notification systems that delay timely data capture. Jia, Liu [84] further emphasised that countries with interoperable digital systems were better equipped to detect outbreaks early and respond effectively. Such findings underscore the value of advancing integrated PHI systems to improve timeliness, coordination, and overall surveillance capacity.

### Strengths and limitations of the review

This review systematically synthesises peer-reviewed studies on PHI tools for dengue risk management, focusing on core operational systems such as EHRs, GIS, HIE, and visualisation platforms. This focus offers a clearer picture of how these technologies support surveillance, prevention, and response than broader scoping reviews that have included machine learning, social media, and mobile applications, such as the Indonesian study by Salim, Satoto [80]. The systematic methodology ensured transparency and reproducibility, and the organising framework of epidemiological insights, mapping and visualisation, and enhanced surveillance provided a practical way to interpret diverse applications.

Several limitations affect this study. This review excluded studies primarily involving predictive or forecasting modelling, mathematical modelling, statistical modelling, AI, or ML were excluded when they were not integrated into operational PHI tools or systems. However, these approaches can contribute to dengue preparedness by supporting risk prediction, early warning, outbreak forecast, and the identification of spatial-temporal risk patterns [77,85]. Their translation into operational public health practice may depend on data availability, data quality, transparency, validation, and integration into public health workflows [86].

The exclusion of grey literature, government reports, and non-English publications may have led to the omission of practical evidence on PHI implementation, particularly where surveillance systems are documented outside indexed English-language journals. Grey and non-English literature can contribute to the evidence of systematic reviews, although its influence varies across topics and contexts [87]. The geographical gaps identified in this review reflect limitations in the eligible peer-reviewed English-language literature, rather than absence of PHI tools. It is also likely that routine surveillance systems, dashboards, laboratory reporting platforms, or digital initiatives may be reported locally or internally rather than in academic publications.

In this systematic review a single reviewer conducted the title, abstract, and full-text screening. To partially mitigate this limitation, a random sample of the screened records was independently checked by a senior reviewer, with full agreement reached for the verified records. Because complete independent duplicate screening was not undertaken, the possibility of selection error or reviewer bias cannot be fully excluded and should be considered when interpreting the comprehensiveness of the provided evidence base [88,89].

The included studies were methodologically diverse, varying in study design, data sources, geographical scale, study period, PHI tool type, and outcome definitions. This heterogeneity limited direct comparison across studies and prevented quantitative synthesis. A narrative synthesis was therefore used to summarise how PHI tools have been reported in dengue risk management, rather than to estimate their pooled effectiveness [90].

## Conclusions

Public health informatics (PHI) tools represent a rapidly evolving technology that requires further research focusing on specific tools, such as GIS, to reduce heterogeneity. There is a need to establish standard criteria for a more comprehensive understanding of their application in controlling and preventing vector-borne diseases like dengue fever. Additionally, standardising outcome measures would help develop uniform metrics, such as outbreak detection time, response efficiency, and improvements in intervention strategies. Establishing these consistent evaluation criteria would provide a clearer framework for assessing the effectiveness of PHI tools in disease surveillance and control.

In conclusion, this review shows that public health informatics tools make an important but uneven contribution to dengue surveillance and control. GIS has been the most widely applied, while HIE remain underutilised. The evidence highlights clear gaps, including limited standardised evaluation measures and underrepresentation of several high-risk regions. Addressing these gaps will require greater integration of PHI systems, investment in digital capacity, and the adoption of common metrics to ensure their effectiveness in strengthening global dengue control efforts.

## Supporting information

S1 PRISMA Checklist PRISMA 2020 checklist. This file contains the completed 27-item PRISMA 2020 checklist for the reporting of this systematic review. The PRISMA 2020 checklist is reproduced under the terms of the Creative Commons Attribution (CC BY 4.0) license. Page MJ, McKenzie JE, Bossuyt PM, et al. The PRISMA 2020 statement: an updated guideline for reporting systematic reviews. BMJ. 2021;372:n71.
(DOCX)

S1 Table Eligibility assessment and inclusion or exclusion decisions for full-text articles reviewed. This table presents the full-text articles assessed during the eligibility stage of the systematic review. For each article, bibliographic details, country of study, and the final inclusion or exclusion decision are reported. Where articles were excluded, the specific exclusion criterion applied is stated, based on the predefined eligibility criteria of the review.
(DOCX)

S2 Table Summary of data extracted from included studies in the systematic review. This table presents the mandatory reproducibility information for all included studies, including citation details, eligibility confirmation, data extractor and extraction dates, study design and methodology, type of study, data sources, public health informatics (PHI) tools used, study aims, participant characteristics, study period, geographic coverage, extracted outcomes, key findings, and authors’ conclusions.
(DOCX)

S3 Table GRADE Evidence Profile for Public Health Informatics Tools in Dengue Fever Surveillance, Prevention, and Control. All three categories were rated very low certainty, reflecting observational study designs, methodological heterogeneity, small evidence bases in two of the three categories, and the uniform reporting of positive tool performance across all included studies. Certainty was assessed using the GRADE framework across five domains: risk of bias, inconsistency, indirectness, imprecision, and publication bias.
(DOCX)
