# Supplementary material for: Public health informatics tools for dengue risk management: A systematic review
Source: PLOS Digit Health. 2026 Jul 9;5(7):e0001495. doi: 10.1371/journal.pdig.0001495 (PMC13349116; doi:10.1371/journal.pdig.0001495)
Supplement: S1 PRISMA Checklist — This file contains the completed 27-item PRISMA 2020 checklist for the reporting of this systematic review. The PRISMA 2020 checklist is reproduced under the terms of the Creative Commons Attribution (CC BY 4.0) license. Page MJ, McKenzie JE, Bossuyt PM, et al. The PRISMA 2020 statement: an updated guideline for reporting systematic reviews. BMJ. 2021;372:n71. (DOCX) [file pdig.0001495.s001.docx]

**S1 PRISMA Checklist.** PRISMA 2020 checklist. This file contains the completed 27-item PRISMA 2020 checklist for the reporting of this systematic review.

| Section and Topic | Item # | Checklist item | Location where item is reported |
| --- | --- | --- | --- |
| **TITLE** | | |  |
| Title | 1 | Identify the report as a systematic review. | Title page |
| **ABSTRACT** | | |  |
| Abstract | 2 | See the PRISMA 2020 for Abstracts checklist. | Abstract section |
| **INTRODUCTION** | | |  |
| Rationale | 3 | Describe the rationale for the review in the context of existing knowledge. | Introduction, Paragraph 1–2 |
| Objectives | 4 | Provide an explicit statement of the objective(s) or question(s) the review addresses. | Introduction, last paragraph; Abstract |
| **METHODS** | | |  |
| Eligibility criteria | 5 | Specify the inclusion and exclusion criteria for the review and how studies were grouped for the syntheses. | Methods, “Eligibility Criteria” section |
| Information sources | 6 | Specify all databases, registers, websites, organisations, reference lists and other sources searched or consulted to identify studies. Specify the date when each source was last searched or consulted. | Methods, Search Strategy’ section |
| Search strategy | 7 | Present the full search strategies for all databases, registers and websites, including any filters and limits used. | Methods, “Search Strategy” section |
| Selection process | 8 | Specify the methods used to decide whether a study met the inclusion criteria of the review, including how many reviewers screened each record and each report retrieved, whether they worked independently, and if applicable, details of automation tools used in the process. | “Title and abstract screening were conducted by a single reviewer against pre-defined inclusion and exclusion criteria, with 30% of screened records independently verified by a second reviewer. Discrepancies were resolved by consensus. No automation tools were used.”  Methods, "Inclusion and Exclusion Criteria" section; Search Strategy subsection |
| Data collection process  Data items | 9 | Specify the methods used to collect data from reports, including how many reviewers collected data from each report, whether they worked independently, any processes for obtaining or confirming data from study investigators, and if applicable, details of automation tools used in the process. | Methods, “Quality appraisal, risk of bias, reporting bias, and data extraction”; Supporting Information, Table S3. |
| Data collection process | 10a | List and define all outcomes for which data were sought. Specify whether all results that were compatible with each outcome domain in each study were sought (e.g. for all measures, time points, analyses), and if not, the methods used to decide which results to collect. | Methods, “Quality appraisal, risk of bias, reporting bias, and data extraction”; Supporting Information, Table S3. Key extracted outcomes are summarised in Table 4. |
|  | 10b | List and define all other variables for which data were sought (e.g. participant and intervention characteristics, funding sources). Describe any assumptions made about any missing or unclear information. | Methods, “Quality appraisal, risk of bias, reporting bias, and data extraction”; Supporting Information, Table S3. Study characteristics are summarised in Table 4. |
| Study risk of bias assessment | 11 | Specify the methods used to assess risk of bias in the included studies, including details of the tool(s) used, how many reviewers assessed each study and whether they worked independently, and if applicable, details of automation tools used in the process. | Methods, “Quality appraisal, risk of bias, reporting bias, and data extraction”. Risk of bias and quality appraisal were assessed using the Newcastle-Ottawa Scale (NOS), including adapted NOS criteria for cross-sectional studies. Assessment domains included selection, comparability, and outcome assessment. |
| Effect measures | 12 | Specify for each outcome the effect measure(s) (e.g. risk ratio, mean difference) used in the synthesis or presentation of results. | Methods, “Data Synthesis”. This review used a narrative synthesis approach. No statistical effect measures were applied. |
| Synthesis methods | 13a | Describe the processes used to decide which studies were eligible for each synthesis (e.g. tabulating the study intervention characteristics and comparing against the planned groups for each synthesis (item #5)). | Methods, “Data Synthesis”. |
|  | 13b | Describe any methods required to prepare the data for presentation or synthesis, such as handling of missing summary statistics, or data conversions. | Methods, “Data Synthesis”. |
|  | 13c | Describe any methods used to tabulate or visually display results of individual studies and syntheses. | Table 4 summarises the characteristics of included studies. Table 5 presents the NOS quality/risk-of-bias assessment for each included study. Supporting Information Table S3 presents the full data extraction record. |
|  | 13d | Describe any methods used to synthesize results and provide a rationale for the choice(s). If meta-analysis was performed, describe the model(s), method(s) to identify the presence and extent of statistical heterogeneity, and software package(s) used. | Methods, “Data Synthesis”. |
|  | 13e | Describe any methods used to explore possible causes of heterogeneity among study results (e.g. subgroup analysis, meta-regression). | Methods, 'Data Synthesis' |
|  | 13f | Describe any sensitivity analyses conducted to assess robustness of the synthesized results. | Not applicable |
| Reporting bias assessment | 14 | Describe any methods used to assess risk of bias due to missing results in a synthesis (arising from reporting biases). | Reporting bias was assessed qualitatively by comparing each study's stated objectives against its published results. At the synthesis level, publication bias was evaluated as part of the GRADE certainty assessment by examining the direction of findings across included studies. Formal statistical tests were not conducted as studies reported descriptive outcomes not amenable to quantitative synthesis. "Reporting Bias" section; Method, "Certainty of Evidence" Methods. |
| Certainty assessment | 15 | Describe any methods used to assess certainty (or confidence) in the body of evidence for an outcome. | The GRADE framework was applied to three functional categories, each assessed across five domains: risk of bias, inconsistency, indirectness, imprecision, and publication bias, classified as high, moderate, low, or very low.  “Certainty of Evidence”, Methods, S4 Table. |
| **RESULTS** | | |  |
| Study selection | 16a | Describe the results of the search and selection process, from the number of records identified in the search to the number of studies included in the review, ideally using a flow diagram. | Results, Figure 1 PRISMA flow diagram |
|  | 16b | Cite studies that might appear to meet the inclusion criteria, but which were excluded, and explain why they were excluded. | Results, Figure 1 PRISMA flow diagram |
| Study characteristics | 17 | Cite each included study and present its characteristics. | Results, Table 4; Supporting Information, Table S3; narrative synthesis categories in the Results section. |
| Risk of bias in studies | 18 | Present assessments of risk of bias for each included study. | Results, “Risk of bias, quality appraisal, and interpretation of evidence”; Table 5. Study-level NOS assessments are presented for each included study. |
| Results of individual studies | 19 | For all outcomes, present, for each study: (a) summary statistics for each group (where appropriate) and (b) an effect estimate and its precision (e.g. confidence/credible interval), ideally using structured tables or plots. | Results, Table 4; Supporting Information, Table S3; narrative synthesis categories in the Results section. No pooled effect estimates were calculated because the review used narrative synthesis. |
| Results of syntheses | 20a | For each synthesis, briefly summarise the characteristics and risk of bias among contributing studies. | Study characteristics are presented in Table 4 and Supporting Information Table S3. Risk-of-bias findings are presented in Table 5 and summarised in Results, “Risk of bias, quality appraisal, and interpretation of evidence”. |
|  | 20b | Present results of all statistical syntheses conducted. If meta-analysis was done, present for each the summary estimate and its precision (e.g. confidence/credible interval) and measures of statistical heterogeneity. If comparing groups, describe the direction of the effect. | No statistical meta-analysis was conducted. Results were synthesised narratively and organised into three categories: epidemiological insights, mapping and visualisation, and enhanced surveillance. |
|  | 20c | Present results of all investigations of possible causes of heterogeneity among study results. | No formal subgroup analysis or meta-regression was conducted. Heterogeneity was considered narratively through differences in study design, PHI tools, data sources, geographic settings, and reported outcomes. |
|  | 20d | Present results of all sensitivity analyses conducted to assess the robustness of the synthesized results. | No sensitivity analyses were conducted because no meta-analysis was performed. |
| Reporting biases | 21 | Present assessments of risk of bias due to missing results (arising from reporting biases) for each synthesis assessed. | Publication bias was assessed as part of the GRADE certainty of evidence evaluation for each functional category. All three categories were downgraded for suspected publication bias, reflecting the uniform reporting of positive findings across all 19 included studies. Formal statistical tests were not conducted as the included studies reported descriptive outcomes not amenable to quantitative synthesis. Result and discussion sections. |
| Certainty of evidence | 22 | Present assessments of certainty (or confidence) in the body of evidence for each outcome assessed. | Certainty of evidence was assessed using the GRADE framework applied to three functional categories across five domains: risk of bias, inconsistency, indirectness, imprecision, and publication bias. All three categories were rated as very low certainty. See Methods and Results. |
| **DISCUSSION** | | | |
| Discussion | 23a | Provide a general interpretation of the results in the context of other evidence. | Discussion section |
|  | 23b | Discuss any limitations of the evidence included in the review. | Discussion, Strengths and limitations. |
|  | 23c | Discuss any limitations of the review processes used. | Discussion, Strengths and limitations. |
|  | 23d | Discuss implications of the results for practice, policy, and future research. | Discussion, Geographical diversity, and Recommendations. |
| **OTHER INFORMATION** | | | |
| Registration and protocol | 24a | Provide registration information for the review, including register name and registration number, or state that the review was not registered. | Methods |
|  | 24b | Indicate where the review protocol can be accessed, or state that a protocol was not prepared. | Protocol registered on PROSPERO |
|  | 24c | Describe and explain any amendments to information provided at registration or in the protocol. | No amendments reported |
| Support | 25 | Describe sources of financial or non-financial support for the review, and the role of the funders or sponsors in the review. | No funding or sponsorship was received for this review. |
| Competing interests | 26 | Declare any competing interests of review authors. | Declarations section. The authors declare no competing interests. |
| Availability of data, code and other materials | 27 | Report which of the following are publicly available and where they can be found: template data collection forms; data extracted from included studies; data used for all analyses; analytic code; any other materials used in the review. | Supporting Information. The full data extraction record is provided in Table S3, including citation details, eligibility confirmation, data extractor and extraction dates, study design, data sources, PHI tools used, extracted outcomes, key findings, and authors’ conclusions. No analytic code was used because no meta-analysis or statistical synthesis was conducted. |
