# Supplementary material for: Public health informatics tools for dengue risk management: A systematic review
Source: PLOS Digit Health. 2026 Jul 9;5(7):e0001495. doi: 10.1371/journal.pdig.0001495 (PMC13349116; doi:10.1371/journal.pdig.0001495)
Supplement: S1 Table — This table presents the full-text articles assessed during the eligibility stage of the systematic review. For each article, bibliographic details, country of study, and the final inclusion or exclusion decision are reported. Where articles were excluded, the specific exclusion criterion applied is stated, based on the predefined eligibility criteria of the review. (DOCX) [file pdig.0001495.s002.docx]

**S1 Table. Eligibility assessment and inclusion or exclusion decisions for full-text articles reviewed**. This table presents the full-text articles assessed during the eligibility stage of the systematic review. For each article, bibliographic details, country of study, and the final inclusion or exclusion decision are reported. Where articles were excluded, the specific exclusion criterion applied is stated, based on the predefined eligibility criteria of the review.

| NO | Title of Article | Author(s) | Year | Country | Include/Exclude | Reason for Exclusion |
| --- | --- | --- | --- | --- | --- | --- |
| 1 | Impact of storm drains on the maintenance of dengue endemicity in Presidente Prudente, São Paulo, Brazil: a geospatial and epidemiologic approach | Bertacco E.A.M. et al. | 2024 | Brazil | Excluded | Study focused on animals or just the vectors (mosquitoes). |
| 2 | Space-time dispersion of dengue occurrence in epidemic and non-epidemic years in a municipality in the metropolitan region of Belo Horizonte, MG, 2011 to 2017 | Sousa S.C. et al. | 2024 | Brazil | Excluded | Study focused on spatial and spatio-temporal epidemiological analysis of dengue incidence using GIS and statistical tools (Moran’s index, SaTScan, incidence smoothing) to describe disease patterns, without assessment of public health informatics tools. |
| 3 | Dengue in the urban slums of Pakistan: health costs, adaptation practices, and the role of dengue-diagnosis and surveillance in controlling the epidemic | Mehmood Y; Arshad M | 2024 | Pakistan | Excluded | Studies focused on health communication and social media tools only. |
| 4 | Spatial mapping of dengue fever prevalence and its association with geo-climatic factors in Lahore, Pakistan | Wafa Rehman, Muhammad Nasar-u-Minallah, Ibtisam Butt | 2024 | Pakistan | Included |  |
| 5 | Epidemiological data monitoring dashboards as a surveillance and healthcare management strategy | Ferraz VCAB et al. | 2024 | Brazil | Excluded | Studies that used public health informatics tools for predicting and forecasting the next outbreak. |
| 6 | Weather integrated multiple machine learning models for prediction of dengue prevalence in India | Kakarla et al. | 2023 | India | Excluded | Study focused on statistical and analytical modelling methods that used public health informatics tools for predicting and forecasting the next outbreak |
| 7 | WebGIS-Based Real-Time Surveillance and Response System for Vector-Borne Infectious Diseases | Javaid et al. | 2023 | Pakistan | Excluded | Study focused on statistical and analytical modelling methods; Articles that focused on infectious diseases or vector borne diseases other than dengue fever; Studies that used public health informatics tools for predicting and forecasting the next outbreak |
| 8 | Dengue in Timor-Leste during the COVID-19 phenomenon | Zito Viegas da Cruz, Afonso Lima Araujo, Alexis Ribas, Choosak Nithikathkul | 2023 | Timor-Leste | Include |  |
| 9 | Assessing dengue fever risk in Costa Rica by using climate variables and machine learning techniques | Barboza et al. | 2023 | Costa Rica | Excluded | Study focused on statistical and analytical modelling methods that used public health informatics tools for predicting and forecasting the next outbreak |
| 10 | PICTUREE—Aedes: A Web Application for Dengue Data Visualization and Case Prediction | Yi C. et al. | 2023 | Multi-country | Excluded | Studies that used public health informatics tools to predict and forecast future outbreaks, and articles focused on mathematical modelling and algorithms with an emphasis on mosquito populations. |
| 11 | Application of medical information system to identify dengue outbreak factors: Insights from a hyperendemic city in Malaysia | Ng et.al. | 2023 | Malaysia | Included |  |
| 12 | Data-driven computational intelligence applied to dengue outbreak forecasting: a case study at the scale of the city of Natal, RN-Brazil | Sánchez-Gendriz et al. | 2022 | Brazil | Excluded | Study focused on statistical and analytical modelling methods that used public health informatics tools for predicting and forecasting the next outbreak |
| 13 | Machine-Learning–Based Forecasting of Dengue Fever in Brazilian Cities Using Epidemiologic and Meteorological Variables | Roster K., Connaughton C., Rodrigues F.A. | 2022 | Brazil | Excluded | Study focused on statistical and analytical modelling methods that used public health informatics tools for predicting and forecasting the next outbreak |
| 14 | Deep learning models for forecasting dengue fever based on climate data in Vietnam | Nguyen et al. | 2022 | Vietnam | Excluded | Study focused on statistical and analytical modelling methods that used public health informatics tools for predicting and forecasting the next outbreak |
| 15 | Temporal and spatial patterns of dengue geographical distribution in Jeddah, Saudi Arabia | Al-Nefaie H; Alsultan A; Abusaris R | 2022 | Saudi Arabia | Excluded | Articles focused on statistical methods and GIS-based descriptive or analytical studies without assessment or implementation of public health informatics tools. |
| 16 | A Privacy-Preserved Internet-of-Medical-Things Scheme for Eradication and Control of Dengue Using UAV | Ali et al. | 2022 | Pakistan | Excluded | Articles focused on animals or just the vectors (mosquitoes); Articles focused on Study focused on statistical and analytical modelling methods |
| 17 | Spatial and Temporal Analysis of Dengue Cases in Peninsular Malaysia; A Five-Year Analysis from 2016 to 2020 | Khushairi Muhd Nor et al. | 2022 | Malaysia | Included |  |
| 18 | Predicting Dengue Outbreaks with Explainable Machine Learning | Aleixo et al. | 2022 | Brazil | Excluded | Study focused on statistical and analytical modelling methods that used public health informatics tools for predicting and forecasting the next outbreak |
| 19 | Machine-Learning–Based Forecasting of Dengue Fever in Brazilian Cities Using Epidemiologic and Meteorological Variables | Roster K., Connaughton C., Rodrigues F.A. | 2022 | Brazil | Excluded | Study focused on modelling mathematical modelling and algorithms that used public health informatics tools for predicting and forecasting the next outbreak |
| 20 | Development and Comparison of Dengue Vulnerability Indices Using GIS-Based Multi-Criteria Decision Analysis in Lao PDR and Thailand | Zafar et al. | 2021 | Thailand | Excluded | Study focused on statistical and analytical modelling methods applied to simulated vulnerability indices used to identify priority areas for dengue fever control, as well as comparative weighting methods (WADI, Shannon Entropy, and BWM). |
| 21 | Identification of Significant Climatic Risk Factors and Machine Learning Models in Dengue Outbreak Prediction | Yavari Nejad F., Varathan K.D. | 2021 | Malaysia | Excluded | Study focused on statistical and analytical modelling methods that used public health informatics tools for predicting and forecasting the next outbreak |
| 22 | Multivariate spatio-temporal approach to identify vulnerable localities in dengue risk areas using Geographic Information System (GIS) | Withanage G.P. et al. | 2021 | Sri Lanka | Excluded | Studies that used public health informatics tools for predicting and forecasting the next outbreak |
| 23 | A descriptive analysis of dengue in Peace Corps Volunteers, 2000-2019 | Catherine T. Gulley, Daniel E. Murphy, Scott A. Poe, Kyle Petersen | 2021 | Multi-country | Included |  |
| 24 | Combination of Univariate Long-Short Term Memory Network and Wavelet Transform for Predicting Dengue Case Density in the National Capital Region, the Philippines | Necesito I.V. et al. | 2021 | Philippines | Excluded | Study focused on statistical and analytical modelling methods that used public health informatics tools for predicting and forecasting the next outbreak |
| 25 | A dynamic, ensemble learning approach to forecast dengue fever epidemic years in Brazil using weather and population susceptibility cycles | McGough S.F. et al. | 2021 | Brazil | Excluded | Study focused on statistical and analytical modelling methods that used public health informatics tools for predicting and forecasting the next outbreak |
| 26 | Profile of hospitalization and death records associated to dengue and severe dengue in Minas Gerais between 2000 and 2015 from the Brazilian Public Health System perspective | Gabriel Henrique da Silva et al. | 2021 | Brazil | Included |  |
| 27 | Visual design for a mobile pandemic map system for public health | Lwin M.O. et al. | 2021 | Sri Lanka | Excluded | Studies focused on health communication and social media tools only |
| 28 | Spatial and temporal analysis of hospitalized dengue patients in Bandung: demographics and risk | Lia Faridah et al. | 2021 | Indonesia | Included |  |
| 29 | Association between climate variables and dengue incidence in Nakhon Si Thammarat Province, Thailand | Abdulsalam F.I. et al. | 2021 | Thailand | Excluded | Articles focused on mathematical modelling and algorithms that used predictive modelling approaches to estimate dengue incidence |
| 30 | Evaluation of Neighborhood Socio-Economic Status, as Measured by the Delphi Method, on Dengue Fever Distribution in Jeddah City, Saudi Arabia | Alkhaldy I. ; Barnett P. | 2021 | Saudi Arabia | Excluded | Articles focused on mathematical modelling was used for retrospective spatial analysis only, with no implementation or evaluation of a public health informatics system for dengue surveillance or control. |
| 31 | Time Series Clustering to Improve Dengue Cases Forecasting with Deep Learning | Bogado J.V. et al. | 2021 | Paraguay | Excluded | Study focused on statistical and analytical modelling methods that used public health informatics tools for predicting and forecasting the next outbreak |
| 32 | Relationship between the Incidence of Dengue Virus Transmission in Traditional Market and Climatic Conditions in Kaohsiung City | Huang C.-H. et al. | 2021 | Taiwan | Excluded | Study focused on statistical and analytical modelling methods |
| 33 | Risk Factors and Predictors of Severe Dengue in Saudi Population in Jeddah, Western Saudi Arabia: A Retrospective Study | Hegazi MA et al. | 2020 | Saudi Arabia | Excluded | Articles focused on statistical methods applied to observational clinical data without use or evaluation of public health informatics tools. |
| 34 | Comparing machine learning with case-control models to identify confirmed dengue cases | Ho T.-S. et al. | 2020 | Taiwan | Excluded | Study focused on statistical and analytical modelling methods applied to prediction and classification of dengue cases. |
| 35 | Assessing the risk of dengue severity using demographic information and laboratory test results with machine learning | Huang S.-W. et al. | 2020 | Taiwan | Excluded | Study focused on statistical and analytical modelling methods |
| 36 | A geo medical analysis of dengue cases in Madurai city-Tamilnadu India | D. Balaji and V. Saravanabavan​ | 2020 | India. | Included |  |
| 37 | Forecast of Dengue Cases in 20 Chinese Cities Based on the Deep Learning Method | Xu J. et al. | 2020 | China | Excluded | Study focused on modelling mathematical modelling and algorithms for forecast dengue cases |
| 38 | A spatial-temporal study for the spread of dengue depending on climate factors in Pakistan (2006–2017) | Shabbir W.; Pilz J.; Naeem A. | 2020 | Pakistan | Excluded | Study focused on statistical and analytical modelling methods |
| 39 | Dengue Hotspot Detection in Bangi, Selangor, Malaysia | Nuriah Abd Majid and Rozimah Muhamad Rasdi​ | 2020 | Malaysia | Included |  |
| 40 | Predicting dengue importation into Europe, using machine learning and model-agnostic methods | Salami D. et al. | 2020 | Europe (multi-country) | Excluded | Study focused on modelling mathematical modelling and algorithms |
| 41 | Automatic stratification of priority areas for Dengue control using the QGIS Model Builder in multicriteria analysis | Paiva Júnior E.F. et al. | 2020 | Brazil | Excluded | Study focused on animals or vectors (mosquitoes), rather than dengue disease surveillance or control. |
| 42 | Epidemiological Characteristics of Dengue Fever Outbreaks in China, 2015–2018 | Zhang X. et al. | 2020 | China | Excluded | Study published in a language other than English. |
| 43 | Past, Present, and Future Vulnerability to Dengue in Jamaica: A Spatial Analysis of Monthly Variations | Henry S. ; Mendonça F. de A. | 2020 | Jamaica | Excluded | Study focused on vulnerability and risk assessment (WADI framework) rather than public health informatics tools for dengue surveillance, control, or prevention. |
| 44 | Towards a machine learning-based approach to forecasting Dengue virus outbreaks in Colombian cities: a case-study: Medellin, Antioquia | Ceballos-Arroyo et al. | 2020 | Colombia | Excluded | Study focused on modelling mathematical modelling and algorithms for forecast dengue cases |
| 45 | Spatiotemporal patterns and climatic drivers of severe dengue in Thailand | Xu Z. et al. | 2019 | Thailand | Excluded | Study focused on statistical and analytical modelling methods |
| 46 | Renal Manifestations of Dengue Viral Infection | Eswarappa M. et al. | 2019 | India | Excluded | Evidence is not based on public health informatics tools; study focused on clinical and laboratory characteristics of dengue patients. |
| 47 | Geographic information system based spatio-temporal dengue fever cluster analysis and mapping | Shuchi Mala and Mahesh Kumar Jat | 2019 | India | Included |  |
| 48 | Dynamics and epidemiological characterization of the dengue outbreak in Argentina 2016: the case of the Province of Buenos Aires | Bolzan et al. | 2019 | Argentina | Excluded | Study published in a language other than English. |
| 49 | The addition of mobile SMS effectively improves dengue prevention practices in community: an implementation study in Nepal | Bhattarai A.H. et al. | 2019 | Nepal | Excluded | Study focused on health communication tools (SMS-based intervention) only. |
| 50 | Spatial and temporal analysis of dengue infections in Queensland, Australia: Recent trend and perspectives | Akter, R. et al. | 2019 | Australia | Included |  |
| 51 | Spatiotemporal dengue fever hotspots associated with climatic factors in Taiwan including outbreak predictions based on machine-learning | Anno S. et al. | 2019 | Taiwan | Excluded | Study focused on modelling mathematical modelling and algorithms applied to simulation and outbreak prediction studies. |
| 52 | Dengue hospitalisations in Brazil: annual wave from West to East and recent increase among children | Wunderlich J.; Acuña-Soto R.; Alonso W.J. | 2018 | Brazil | Excluded | Study focused on statistical and analytical modelling methods applied to analytical studies. |
| 53 | Spatial distribution and cluster analysis of dengue using self-organizing maps in Andhra Pradesh, India, 2011–2013 | Mutheneni S.R. et al. | 2018 | India | Excluded | Study focused on statistical and analytical modelling methods applied to retrospective epidemiological data, without implementation or evaluation of public health informatics systems for dengue surveillance, control, or prevention. |
| 54 | A Gaussian process based big data processing framework in cluster computing environment | Manogaran G.; Lopez D. | 2018 | India | Excluded | Study focused on statistical methods applied to prediction and forecasting of dengue outbreaks using climate and environmental data. |
| 55 | Investigating spatio-temporal distribution and diffusion patterns of the dengue outbreak in Swat, Pakistan | Atique, S. et al. | 2018 | Pakistan | Included |  |
| 56 | Surveillance of intensity level and geographical spreading of dengue outbreak among males and females in Punjab, Pakistan: A case study of 2011 | Ahmad, S. et al. | 2018 | Pakistan | Included |  |
| 57 | A model comparison algorithm for increased forecast accuracy of dengue fever incidence in Singapore and the auxiliary role of total precipitation information | Koh Y.-M. et al. | 2018 | Singapore | Excluded | Study focused on statistical and analytical modelling methods (e.g., time-series models and neural networks) for forecasting dengue incidence using climatic data. |
| 58 | Determination of environmental factors affecting dengue incidence in Sleman District, Yogyakarta, Indonesia | Kesetyaningsih T.W. et al. | 2018 | Indonesia | Excluded | Study focused on statistical and GIS-based analytical methods to examine associations between environmental and climatic factors and dengue incidence, without implementation or evaluation of a public health informatics system for dengue surveillance, control, or prevention. |
| 59 | Machine learning methods reveal the temporal pattern of dengue incidence using meteorological factors in metropolitan Manila, Philippines | Carvajal T.M. et al. | 2018 | Philippines | Excluded | Study focused on statistical methods for predicting and forecasting dengue incidence using meteorological data. |
| 60 | Dengue forecasting in São Paulo city with generalized additive models, artificial neural networks and seasonal autoregressive integrated moving average models | Baquero O.S. et al. | 2018 | Brazil | Excluded | Study focused on statistical and analytical modelling methods (e.g., time-series models and artificial neural networks) for predicting and forecasting dengue incidence using meteorological data. |
| 61 | Advances in using Internet searches to track dengue | Yang S.; Kou S.C. et al. | 2017 | Multiple countries (Mexico, Brazil, Thailand, Singapore, Taiwan) | Excluded | Study focused on statistical and analytical modelling methods using Internet search data (e.g., Google Trends) to estimate and predict dengue activity, without implementation or evaluation of a public health informatics surveillance system for dengue control or prevention. |
| 62 | Effect of Climatic Factors and Population Density on the Distribution of Dengue in Sri Lanka: A GIS Based Evaluation for Prediction of Outbreaks | Sirisena P.D.N.N. et al. | 2017 | Sri Lanka | Excluded | Study focused on statistical and GIS-based analytical and time-series modelling methods for predicting and forecasting dengue outbreaks using climatic and population data. |
| 63 | Dengue Baidu Search Index data can improve the prediction of local dengue epidemic: A case study in Guangzhou, China | Li Z. et al. | 2017 | China | Excluded | Study focused on statistical and analytical modelling methods using internet search query data and generalized additive models to predict and forecast dengue outbreaks. |
| 64 | 20 Years Spatial-Temporal Analysis of Dengue Fever and Hemorrhagic Fever in Mexico | Hernández-Gaytán S.I. et al. | 2017 | Mexico | Excluded | Study focused on statistical and GIS-based spatial and space-time analytical methods to describe dengue distribution and clustering, without implementation or evaluation of a public health informatics system for dengue surveillance, control, or prevention. |
| 65 | The Application Geographic Information System Dengue Haemorrhagic Fever Risk Assessment in Samut Songkhram Province, Thailand | Chaiphongpachara, T. et al. | 2017 | Thailand | Included |  |
| 66 | Can internet search queries be used for dengue fever surveillance in China? | Guo P. et al. | 2017 | China | Excluded | Study focused on statistical and analytical modelling methods using internet search query data to estimate and predict dengue activity. |
| 67 | Information Technology and Public Health: Possibilities for Innovation through Interdisciplinary Actions | de Camargo J.T.F. et al. | 2017 | Brazil | Excluded | Study focused on animals or vectors (Aedes aegypti) and vector breeding-site monitoring. |
| 68 | Analysis of the spatial distribution of dengue cases in the city of Rio de Janeiro, 2011 and 2012 | Carvalho S. et al. | 2017 | Brazil | Excluded | Study focused on GIS-based spatial and statistical analytical methods to describe the distribution of dengue cases. |
| 69 | Exploring the Role of Syndromic Surveillance in the Early Detection of Disease Outbreaks | Liu X. et al. | 2016 | China | Excluded | Study published in a language other than English. |
| 70 | Sensitivity of the Dengue Surveillance System in Brazil for Detecting Hospitalized Cases | Coelho, G. E. et al. | 2016 | Brazil | Included |  |
| 71 | The Spread of Dengue in an Endemic Urban Milieu – The Case of Delhi, India | Telle O. et al. | 2016 | India | Excluded | Study focused on analysing the relationship between population density and socio-economic and urban conditions associated with dengue transmission, using statistical and spatial analytical methods, without implementation or evaluation of public health informatics tools for dengue surveillance, control, or prevention. |
| 72 | Mapping intra-urban transmission risk of dengue fever with big hourly cellphone data | Mao L. et al. | 2016 | China | Excluded | Study focused on statistical and analytical modelling methods using big data (cellphone tracking data, machine learning, and GIS) to assess and predict dengue transmission risk. |
| 73 | M-DENGUE: Utilizing Crowdsourcing and Teleconsultation for Location-Based Dengue Monitoring and Reporting System | Husain W. et al. | 2016 | Malaysia | Excluded | Study focused on health communication and participatory reporting tools (crowdsourcing, teleconsultation, and public reporting platforms). |
| 74 | Geographical Information System Based Approach to Monitor Epidemiological Disaster: 2011 Dengue Fever Outbreak in Punjab, Pakistan | Ahmad, S. et al. | 2016 | Pakistan | Included |  |
| 75 | Spatio-Temporal Extension and Spatial Analyses of Dengue from Rawalpindi, Islamabad and Swat during 2010–2014 | Fareed N. et al. | 2016 | Pakistan | Excluded | Study focused on statistical, spatial, and spatio-temporal analytical modelling using GIS and remote sensing to examine associations between climatic, environmental, and socio-demographic factors and dengue incidence. |
| 76 | Temporal Dynamics and Spatial Patterns of Aedes aegypti Breeding Sites, in the Context of a Dengue Control Program in Tartagal (Salta Province, Argentina) | Espinosa M. et al. | 2016 | Argentina | Excluded | Study focused exclusively on entomological surveillance and spatial analysis of Aedes aegypti breeding sites using GIS, remote sensing, and ecological modelling. |
| 77 | DengueME: A Tool for the Modeling and Simulation of Dengue Spatiotemporal Dynamics | Lima T.F.M. et al. | 2016 | Brazil | Excluded | Study focused on statistical and computational modelling and simulation frameworks for dengue and vector dynamics. |
| 78 | Online platform for applying space–time scan statistics for prospectively detecting emerging hot spots of dengue fever | Chen C.-C. et al. | 2016 | Taiwan | Excluded | Study focused on an online analytical platform using statistical modelling to prospectively detect dengue transmission. |
| 79 | Dengue incidence trend in Brazil, 2002–2012 | Böhm A.W. et al. | 2016 | Brazil | Excluded | Study focused on descriptive epidemiological and time-series trend analysis of dengue incidence using routine surveillance data, without assessment or application of EHR-based public health informatics tools, GIS, HIE, or data visualisation systems for dengue surveillance, control, or prevention. |
| 80 | Risk analysis for dengue suitability in Africa using the ArcGIS predictive analysis tools | Attaway D.F. et al. | 2016 | Multiple countries (Africa) | Excluded | Study focused on GIS-based predictive and suitability modelling using environmental and climatic data to map dengue risk. |
| 81 | Dengue Outbreak Prediction for GIS-Based Early Warning System | Tazkia R.A.K.; Narita V.; Nugroho A.S. | 2015 | Indonesia | Excluded | Study focused on GIS-based early warning and predictive modelling using data mining (Naïve Bayes) to forecast dengue outbreaks, rather than assessment or application of EHR-based public health informatics tools, HIE, or standalone data visualisation systems. |
| 82 | Predictive Model of Dengue Focus Applied to Geographic Information Systems | Báez González M.; González Rodas G. | 2015 | Paraguay | Excluded | Study focused on predictive and simulation-based modelling of Aedes aegypti population dynamics and dengue risk using GIS and mathematical models. |
| 83 | Spatial distribution and physicochemical characterization of the breeding habitats of Aedes aegypti in and around Kolkata, West Bengal, India | Chatterjee S.; Chakraborty A.; Sinha S.K. | 2015 | India | Excluded | Study focused exclusively on entomological surveillance and ecological analysis of Aedes aegypti breeding habitats using GIS and physicochemical measurements. |
| 84 | Spatial Distribution of Epidemiological Cases of Dengue Fever in Suriname, 2001-2012 | Hamer, D.; Lichtveld, M.; | 2015 | Suriname | Included |  |
| 85 | Web platform using digital image processing and geographic information system tools: a Brazilian case study on dengue | Brasil L.M. et al. | 2015 | Brazil | Excluded | Study focused on entomological surveillance using digital image processing and GIS to count Aedes aegypti eggs from ovitraps via a web platform. |
| 86 | Space–time clustering characteristics of dengue based on ecological, socio-economic and demographic factors in northern Sri Lanka | Anno S. et al. | 2015 | Sri Lanka | Excluded | Study focused on statistical, spatial, and spatio-temporal analytical modelling using GIS and remote sensing to examine associations between ecological, socio-economic, and demographic factors and dengue incidence. |
| 87 | Spatial density of Aedes distribution in urban areas: A case study of Breteau index in Kuala Lumpur, Malaysia | Aziz S. et al. | 2014 | Malaysia | Excluded | Study focused exclusively on entomological surveillance and spatial analysis of Aedes mosquito breeding indices (Breteau index) using GIS, remote sensing, and spatial statistical methods. |
| 88 | Mining Epidemiological Dengue Fever Data from Brazil: A Gradual Pattern Based Geographical Information System | Aryadinata Y.S. et al. | 2014 | Brazil | Excluded | Study focused on climate factors, data mining and GIS-based analytical methods (gradual pattern mining) to explore and visualise epidemiological associations in dengue data, without assessment of public health informatics tools or operational surveillance systems for dengue control or prevention. |
| 89 | Characterization of the Temporal and Spatial Dynamics of the Dengue Epidemic in Northern Sri Lanka | Anno S. et al. | 2014 | Sri Lanka | Excluded | Study focused on analysing climate, ecological, socio-economic, and demographic factors using remote sensing, GIS, and spatial–temporal analytical methods to characterise dengue patterns and inform future modelling. |
| 90 | Are we modelling the correct dataset? Minimizing false predictions for dengue fever in Thailand | Aguiar M. et al. | 2014 | Thailand | Excluded | Study focused on mathematical and statistical modelling, simulation, and predictability analysis of dengue incidence using compartmental models to assess data quality and forecasting performance. |
| 91 | The Mosquito Online Advanced Analytic Service: A Case Study for School Research Projects in Thailand | Wongkoon S. et al. | 2013 | Thailand | Excluded | Study focused on the development of a web-based analytical and educational platform for entomological (mosquito larval) data entry, analysis, and visualisation using GIS and statistical tools. |
| 92 | Cell Phone-Based System (Chaak) for Surveillance of Immatures of Dengue Virus Mosquito Vectors | Lozano-Fuentes S. et al. | 2013 | Mexico | Excluded | Study focused on animals or just the vectors (mosquitoes). |
| 93 | A WebGIS tool for visualizing and exploring socioeconomic vulnerability to dengue fever in Cali, Colombia | Kienberger S. et al. | 2013 | Colombia | Excluded | Study focused on climate and socio-economic vulnerability assessment using WebGIS-based analytical and visualization tools. |
| 94 | Measurement of dengue epidemic spreading pattern using density analysis method: retrospective spatial statistical study of dengue in Subang Jaya, Malaysia, 2006-2010 | Nazri Che Dom. et al. | 2013 | Malaysia | Included |  |
| 95 | Developing a Vulnerability Mapping Methodology: Applying the Water-Associated Disease Index to Dengue in Malaysia | Dickin S.K. et al. | 2013 | Malaysia | Excluded | Study focused on vulnerability and risk assessment using climate, environmental, and socio-economic indicators integrated through GIS (WADI framework) to map dengue risk. |
| 96 | Assessing the risk for dengue fever based on socioeconomic and environmental variables in a geographical information system environment | Khormi H.M.; Kumar L. | 2012 | Saudi Arabia | Excluded | Study focused on climate, environmental, and socio-economic risk assessment using GIS-based analytical and modelling methods to characterise dengue risk. |
| 97 | Risk mapping of dengue in Selangor and Kuala Lumpur, Malaysia | Hassan H.; Shohaimi S.; Hashim N.R. | 2012 | Malaysia | Excluded | Study focused on climate and population factors using GIS-based analytical and geostatistical methods (risk mapping and co-kriging) to model dengue risk, without assessment of public health informatics tools. |
| 98 | Modeling dengue fever risk based on socioeconomic parameters, nationality and age groups: GIS and remote sensing based case study | Khormi H.M.; Kumar L. | 2011 | Saudi Arabia | Excluded | Study focused on socioeconomic risk modelling and prediction of dengue using GIS, remote sensing, and Geographically Weighted Regression (GWR), aiming to identify populations at risk, without assessment of public health informatics tools. |
| 99 | Spatio-temporal diffusion pattern and hotspot detection of dengue in Chachoengsao province, Thailand | Jeefoo, Phaisarn. et al. | 2011 | Thailand | Included |  |
