# Supplementary material for: Public health informatics tools for dengue risk management: A systematic review
Source: PLOS Digit Health. 2026 Jul 9;5(7):e0001495. doi: 10.1371/journal.pdig.0001495 (PMC13349116; doi:10.1371/journal.pdig.0001495)
Supplement: S2 Table — This table presents the mandatory reproducibility information for all included studies, including citation details, eligibility confirmation, data extractor and extraction dates, study design and methodology, type of study, data sources, public health informatics (PHI) tools used, study aims, participant characteristics, study period, geographic coverage, extracted outcomes, key findings, and authors’ conclusions. (DOCX) [file pdig.0001495.s003.docx]

**S2 Table. Summary of data extracted from included studies in the systematic review.** This table presents the mandatory reproducibility information for all included studies, including citation details, eligibility confirmation, data extractor and extraction dates, study design and methodology, type of study, data sources, public health informatics (PHI) tools used, study aims, participant characteristics, study period, geographic coverage, extracted outcomes, key findings, and authors’ conclusions.

| Study Number | Full Citation (Authors, Year, Title, Journal, DOI) | Data extractor & Extraction data | Eligibility Confirmed | Study Design | Methodology | Type of Study | Data Source & Type | PHI Tools Used | Aim / Objective | Population / Participants | Study Period | Geographic Coverage | Outcome Data Extracted | Key Findings | Authors’ Conclusions |
| --- | --- | --- | --- | --- | --- | --- | --- | --- | --- | --- | --- | --- | --- | --- | --- |
| 1 | Rehman W., Nasar-u-Minallah M., Butt I. (2024). *Spatial mapping of dengue fever prevalence and its association with geo-climatic factors in Lahore, Pakistan.* Environmental Monitoring & Assessment. DOI: 10.1007/s10661-024-12967-7 | Naif Alraihan between Jan-Mar 2025 | Yes | Ecological | Quantitative | Observational | DG Health Services Pakistan surveillance data | GIS | Map outbreaks and identify geo-climatic risk areas | 19,882 confirmed cases | 2021 | Lahore, Pakistan | Case counts; NDVI, NDWI, LST, rainfall | Hotspots in dense urban zones; strong links between LST, water bodies and clustering | GIS reveals environmental determinants and supports targeted interventions |
| 2 | Ng et al. (2023). *Application of medical information system to identify dengue outbreak factors.* Int. J. Medical Informatics. DOI: 10.1016/j.ijmedinf.2023.105162 | Naif Alraihan between July-Sep 2024 | Yes | Retrospective observational | Quantitative | Observational | e-Dengue national surveillance | EHR + GIS (QGIS) | Identify outbreak-driving factors | 18,812 confirmed cases | 2012–2019 | Ipoh, Malaysia | Weather indicators; demographic variables; dengue cases | Rainfall–dengue 1-week lag; hotspots near forest edges; high-density zones highest risk | Information system + GIS improves epidemiological understanding |
| 3 | Zito Viegas da Cruz et al. (2023). *Dengue in Timor-Leste during COVID-19.* Frontiers in Public Health. DOI: 10.3389/fpubh.2023.1057951 | Naif Alraihan between July-Sep 2024 | Yes | Ecological | Quantitative | Descriptive/Observational | HIS/EIS under Ministry of Health | HIS + GIS | Evaluate dengue incidence during COVID-19 | 1,556 cases | 2020–2021 | 13 municipalities, Timor-Leste | Dengue and COVID-19 incidence | Urban areas saw increased dengue; diagnosis complicated by overlapping symptoms | GIS supports identification of high-risk locations |
| 4 | Khushairi Muhd Nor et al. (2022). *Spatial and temporal analysis of dengue in Peninsular Malaysia.* Conference paper | Naif Alraihan between July-Sep 2024 | Yes | Ecological | Quantitative | Observational | MOH Malaysia e-Notifikasi | HIS + GIS | Identify hotspots and temporal trends | 457,968 cases | 2016–2020 | Peninsular Malaysia (91 districts) | Dengue incidence; hotspot mapping | Central region had the highest persistent hotspots | GIS assists targeted surveillance |
| 5 | Gulley C. et al. (2021). *Descriptive analysis of dengue in Peace Corps Volunteers, 2000–2019.* Travel Medicine & ID. DOI: 10.1016/j.tmaid.2021.102125 | Naif Alraihan between July-Sep 2024 | Yes | Descriptive | Quantitative | Descriptive | Peace Corps ESS | EMR | Compare pre- and post-EMR surveillance | 1,764 cases | 2000–2019 | 7 global PCV regions | Case trends; demographics | EMR improved completeness and timeliness; 3-year cycles in incidence | EMR systems improve surveillance robustness |
| 6 | da Silva G.H. et al. (2021). *Hospitalization and death records linked to dengue in Minas Gerais.* J Vector Borne Dis. DOI: 10.4103/0972-9062.321738 | Naif Alraihan between July-Sep 2024 | Yes | Retrospective observational | Quantitative | Descriptive | SINAN + SIH databases | EHR systems | Assess dengue and severe dengue burden | 36,330 individuals | 2000–2015 | Minas Gerais, Brazil | Hospitalizations; deaths; demographic variables | Urban areas and young adults most affected | EHR crucial for understanding long-term dengue burden |
| 7 | Faridah L. et al. (2021). *Spatial-temporal analysis of hospitalized dengue in Bandung.* Trop Med & Health. DOI: 10.1186/s41182-021-00329-9 | Naif Alraihan between July-Sep 2024 | Yes | Retrospective observational | Quantitative | Descriptive | Hospital surveillance system | EHR + GIS | Assess spatial-temporal distribution | 10,573 cases | 2014–2016 | Bandung, Indonesia | Hotspots; demographics | Southern/eastern Bandung highest risk; males and children more affected | GIS valuable for hotspot-based planning |
| 8 | Balaji D., Saravanabavan V. (2020). *Geo-medical analysis of dengue in Madurai.* GeoJournal. DOI: 10.1007/s10708-019-10006-4 | Naif Alraihan between July-Sep 2024 | Yes | Ecological | Quantitative | Observational | Health centres & vector unit | GIS | Map distribution and identify high-risk zones | Dengue cases 2013–2015 | 2013–2015 | Madurai, India | Spatial clustering | High-risk urban pockets identified | GIS effective for identifying priority zones |
| 9 | Majid N.A., Rasdi R.M. (2020). *Dengue hotspot detection in Bangi, Malaysia.* IOP Conference Series | Naif Alraihan between July-Sep 2024 | Yes | Descriptive | Quantitative | Observational | MOH Malaysia | GIS | Detect hotspots for public health action | 4,507 cases | 2016–2019 | Bangi, Malaysia | Hotspot clustering | Hotspots concentrated in highly urbanized areas | GIS beneficial for strategic intervention |
| 10 | Mala S., Jat M.K. (2019). *Spatio-temporal dengue cluster analysis, Delhi.* Egyptian J Remote Sensing. DOI: 10.1016/j.ejrs.2019.08.002 | Naif Alraihan between July-Sep 2024 | Yes | Retrospective observational | Quantitative | Observational | Delhi Municipal Corporation | GIS | Identify clusters and associated factors | 9,483 cases | 2010–2012 | Delhi, India | Clusters; demographic factors; wind speed | Significant clusters found; negative correlation with wind speed | GIS improves epidemic pattern detection |
| 11 | Akter R. et al. (2019). *Spatial & temporal dengue analysis, Queensland.* PLOS ONE. DOI: 10.1371/journal.pone.0220134 | Naif Alraihan between July-Sep 2024 | Yes | Retrospective observational | Quantitative | Observational | Queensland Health | GIS | Identify high-risk areas and temporal change | 1,773 cases | 2010–2015 | Queensland, Australia | Autochthonous vs imported trends | Cairns region major hotspot; expansion into new areas | GIS informs resource allocation |
| 12 | Atique S. et al. (2018). *Spatio-temporal dengue diffusion in Swat, Pakistan.* JIPH. DOI: 10.1016/j.jiph.2017.12.003 | Naif Alraihan between July-Sep 2024 | Yes | Ecological | Quantitative | Observational | District Health Office | GIS | Analyze diffusion pattern and clustering | 9,032 cases | 2013 | Swat District, Pakistan | Clusters; demographic variables | Hotspots in dense southern urban zones; elevation inversely linked to risk | GIS supports outbreak response |
| 13 | Ahmad S. et al. (2018). *Surveillance of dengue outbreak in Punjab, 2011.* JIPH. DOI: 10.1016/j.jiph.2017.10.002 | Naif Alraihan between July-Sep 2024 | Yes | Descriptive | Quantitative | Observational | Punjab district hospitals | GIS | Identify hotspots and outbreak patterns | 21,000 cases | Mar–Dec 2011 | Punjab, Pakistan | Hotspots; age–gender correlations | Peak week 30; males age 30 highest risk | GIS supports early warning |
| 14 | Chaiphongpachara T. et al. (2017). *GIS in DHF risk assessment, Thailand.* Int. J. Geomate. DOI: 10.21660/2017.30.160601 | Naif Alraihan between July-Sep 2024 | Yes | Ecological | Quantitative | Observational | Provincial epidemiology office | GIS | Assess DHF risk and influencing factors | DHF cases 2014–2015 | 2014–2015 | Samut Songkhram, Thailand | Environmental & demographic risk indicators | 9% of province “very high risk” | GIS effective tool for risk stratification |
| 15 | Coelho G. et al. (2016). *Sensitivity of dengue surveillance system in Brazil.* PLOS NTD. DOI: 10.1371/journal.pntd.0004705 | Naif Alraihan between July-Sep 2024 | Yes | Cross-sectional | Quantitative | Observational | SINAN + SIH-SUS | EHR systems | Assess sensitivity of surveillance for hospitalized cases | 71,161 hospitalizations | 2008–2013 | 10 Brazilian capitals | Matched cases; sensitivity estimates | 50.8% sensitivity; major under-reporting | Better integration of HIS required |
| 16 | Ahmad S. et al. (2016). *GIS-based monitoring of 2011 dengue outbreak, Punjab.* IJACSA | Naif Alraihan between July-Sep 2024 | Yes | Descriptive | Quantitative | Observational | Manual hospital reporting | GIS | Monitor spread and outbreak intensity | 21,184 cases | 2011 | Punjab province | Spread intensity; hotspots | GIS revealed high-risk districts | GIS essential for early warning |
| 17 | Hamer D., Lichtveld M. (2015). *Spatial distribution of dengue, Suriname.* West Indian Med J. DOI: 10.7727/wimj.2014.118 | Naif Alraihan between July-Sep 2024 | Yes | Cross-sectional | Quantitative | Observational | Suriname Bureau of Public Health | GIS | Characterize incidence and detect clusters | 2,393 cases | 2001–2012 | Suriname | Case distribution; clusters | Significant clusters in Paramaribo and Wanica | GIS effective for identifying historic clusters |
| 18 | Nazri Che Dom et al. (2013). *Dengue epidemic spread in Subang Jaya, Malaysia.* Trans RSTMH. DOI: 10.1093/trstmh/trt073 | Naif Alraihan between July-Sep 2024 | Yes | Descriptive | Quantitative | Observational | Municipal surveillance system | GIS | Analyze epidemic spread and hotspots | 5,601 cases | 2006–2010 | Subang Jaya, Malaysia | Hotspots; kernel density analysis | Persistent hotspots in dense urban areas | GIS enhances epidemic monitoring |
| 19 | Jeefoo P., Tripathi N., Souris M. (2011). Spatio-temporal diffusion pattern and hotspot detection of dengue in Chachoengsao Province, Thailand. International Journal of Environmental Research and Public Health. DOI: 10.3390/ijerph8010051 | Naif Alraihan between July-Sep 2024 | Yes | Cross-sectional | Quantitative | Observational | Provincial public health office (epidemiological surveillance records) | GIS | Examine spatio-temporal diffusion and identify hotspots | 5,831 reported dengue cases | 1999–2007 | Chachoengsao Province, Thailand | Clustering patterns; hotspot areas; demographic and climatic factors | Dengue cases were strongly clustered spatially and temporally; hotspots corresponded with urbanized zones | GIS and spatial analysis effectively identify diffusion patterns and hotspots to support targeted interventions |
