# Supplementary material for: Public health informatics tools for dengue risk management: A systematic review
Source: PLOS Digit Health. 2026 Jul 9;5(7):e0001495. doi: 10.1371/journal.pdig.0001495 (PMC13349116; doi:10.1371/journal.pdig.0001495)
Supplement: S3 Table — All three categories were rated very low certainty, reflecting observational study designs, methodological heterogeneity, small evidence bases in two of the three categories, and the uniform reporting of positive tool performance across all included studies. Certainty was assessed using the GRADE framework across five domains: risk of bias, inconsistency, indirectness, imprecision, and publication bias. (DOCX) [file pdig.0001495.s004.docx]

**S3 Table. GRADE Evidence Profile for Public Health Informatics Tools in Dengue Fever Surveillance, Prevention, and Control**. All three categories were rated very low certainty, reflecting observational study designs, methodological heterogeneity, small evidence bases in two of the three categories, and the uniform reporting of positive tool performance across all included studies. Certainty was assessed using the GRADE framework across five domains: risk of bias, inconsistency, indirectness, imprecision, and publication bias.

| **Mapping and visualisation** 12 studies | | | | **Very low certainty** |
| --- | --- | --- | --- | --- |
| Starting certainty: **Low** (all observational) → Downgraded by: indirectness (−1), publication bias (−1) | | | | |
| Risk of Bias  **Not serious** | Inconsistency  **Not serious** | Indirectness  **Serious −1** | Imprecision  **Not serious** | Publication Bias  **Suspected −1** |
| **Epidemiological insights** 5 studies | | | | **Very low certainty** |
| Starting certainty: **Low** (all observational) → Downgraded by: inconsistency (−1), indirectness (−1), imprecision (−1), publication bias (−1) | | | | |
| Risk of Bias  **Not serious** | Inconsistency  **Serious −1** | Indirectness  **Serious −1** | Imprecision  **Serious −1** | Publication Bias  **Suspected −1** |
| **Enhanced surveillance** 2 studies | | | | **Very low certainty** |
| Starting certainty: **Low** (all observational) → Downgraded by: inconsistency (−1), indirectness (−1), imprecision (−2), publication bias (−1) | | | | |
| Risk of Bias  **Not serious** | Inconsistency  **Serious −1** | Indirectness  **Serious −1** | Imprecision  **Very serious −2** | Publication Bias  **Suspected −1** |
